# Robust designation of meiotic crossover sites by CDK-2 through phosphorylation of the MutSγ complex

**DOI:** 10.1073/pnas.2117865119

**Published:** 2022-05-16

**Authors:** Jocelyn Haversat, Alexander Woglar, Kayla Klatt, Chantal C. Akerib, Victoria Roberts, Shin-Yu Chen, Swathi Arur, Anne M. Villeneuve, Yumi Kim

**Affiliations:** ^a^Department of Biology, Johns Hopkins University, Baltimore, MD 21218; ^b^Department of Developmental Biology, Stanford University School of Medicine, Stanford, CA 94305; ^c^Department of Genetics, Stanford University School of Medicine, Stanford, CA 94305;; ^d^Department of Genetics, University of Texas M.D. Anderson Cancer Center, Houston, TX 77030

**Keywords:** meiosis, recombination, CDK-2, MSH-5, crossover designation

## Abstract

Crossover formation is essential for proper segregation of homologous chromosomes during meiosis. Here, we show that *Caenorhabditis elegans* cyclin-dependent kinase 2 (CDK-2) partners with cyclin-like protein COSA-1 to promote crossover formation by promoting conversion of meiotic double-strand breaks into crossover–specific recombination intermediates. Further, we identify MutSγ component MSH-5 as a CDK-2 phosphorylation target. MSH-5 has a disordered C-terminal tail that contains 13 potential CDK phosphosites and is required to concentrate crossover–promoting proteins at recombination sites. Phosphorylation of the MSH-5 tail appears dispensable in a wild-type background, but when MutSγ activity is partially compromised, crossover formation and retention of COSA-1 at recombination sites are exquisitely sensitive to phosphosite loss. Our data support a model in which robustness of crossover designation reflects a positive feedback mechanism involving CDK-2–mediated phosphorylation and scaffold-like properties of the MSH5 C-terminal tail, features that combine to promote full recruitment and activity of crossover–promoting complexes.

## Significance

Successful chromosome segregation during meiosis relies on crossover recombination between homologous chromosomes. Meiotic recombination initiates with the formation of numerous DNA double-strand breaks, but only a few are ultimately selected to become crossovers. How this process is regulated to ensure that each homolog pair designates at least one crossover remains poorly understood. Here, we show that the *Caenorhabditis elegans* kinase CDK-2 partners with cyclin-like protein COSA-1 and promotes crossover designation through phosphorylation and activation of the MutSγ complex. Our data support a model in which scaffold-like properties of the MSH-5 C-terminal tail and its CDK-2–mediated phosphorylation combine to promote full recruitment and activity of crossover–promoting complexes, thereby generating positive feedback that contributes to the robustness of crossover designation.

Sexually reproducing organisms rely on proper chromosome segregation during meiosis to produce gametes with a complete genome. During meiotic prophase I, chromosomes pair and undergo crossover recombination with their homologous partners. This process, together with sister chromatid cohesion, leads to the formation of physical linkages between the homologs and enables their separation during meiosis I. Defects in crossover formation are disastrous, leading to miscarriages and congenital disorders, such as Down syndrome (1).

Meiotic recombination initiates with the generation of programmed DNA double-strand breaks (DSBs) by the topoisomerase-like enzyme Spo11 (2). DSBs are resected to yield two 3′-end single-stranded DNA (ssDNA) overhangs, which are rapidly coated by RecA recombinases Dmc1 and Rad51. This nucleoprotein filament then seeks out homology and invades a homologous template, forming a metastable single-end invasion intermediate (D-loop) (3). The invading strand primes DNA synthesis and extends the D-loop. If the extended D-loop is captured by ssDNA on the other side of DSBs in a process known as second-end capture, a double Holliday junction (dHJ) forms (4). While dHJs can be resolved biochemically as either crossovers or non–crossovers (5), during meiosis, the majority of dHJs are specifically resolved as crossovers through the activity of MutLγ (MLH1-MLH3) (6–8) or other structure-selective endonucleases. Although a multitude of DSBs are generated during meiotic prophase, strikingly few are ultimately selected to become crossovers. Early recombination intermediates pare down in pachytene until each homolog pair receives at least one crossover, while the majority of DSBs are repaired as non–crossovers via synthesis-dependent strand annealing (9). However, how meiotic DSBs are chosen to become crossovers remains poorly understood.

Throughout eukaryotes, crossover recombination is primarily controlled by a group of proteins collectively termed “ZMM” (10). Notably, homologs of the yeast RING (Really interesting new gene) domain protein Zip3 [ZHP-1, ZHP-2, ZHP-3, and ZHP-4 in *Caenorhabditis elegans* (11–14), *Drosophila* Vilya and Narya/Nenya (15, 16), Hei10 in *Arabidopsis* (17), and Hei10 and RNF212 in mammals (18, 19)] initially localize as abundant foci or long stretches along the synaptonemal complex (SC) but eventually concentrate at crossover sites in late pachytene (20). These SUMO or ubiquitin ligases appear to promote crossover designation by stabilizing the ZMM proteins at crossover sites while removing them from other recombination intermediates (13, 14, 18, 19, 21, 22). Although many meiotic proteins are shown to be SUMO modified (23), key targets of the Zip3 family proteins remain largely unknown.

The meiosis-specific MutS homologs MSH4 and MSH5 form a heterodimeric MutSγ complex and play essential roles in crossover formation in diverse eukaryotes (24–31). MutSγ localizes to recombination intermediates as numerous foci but ultimately accumulates at sites that are destined to become crossovers (32, 33). Biochemical analyses using recombinant MSH4 and MSH5 have shown that MutSγ recognizes single-end invasion intermediates and HJs in vitro (34, 35). HJs activate the ATP hydrolysis of MutSγ and promote the exchange of bound ADP for ATP, inducing the formation of a sliding clamp that dissociates from HJs (35, 36). By iterative loading and embracing DNA duplexes within a dHJ, MutSγ is thought to stabilize crossover–specific recombination intermediates (33, 35). In addition, MutSγ recruits and activates the resolvase activity of MutLγ, enabling biased processing of dHJs into crossovers during meiosis (37, 38).

A genetic screen in *C. elegans* identified a cyclin-like protein COSA-1 as a component essential for processing meiotic DSBs into crossovers (39). The mammalian ortholog CNTD1 was subsequently identified (40), and both COSA-1 and CNTD1 have been shown to localize to crossover sites (39, 41, 42). In the absence of COSA-1/CNTD1, MutSγ components persist as numerous foci in pachytene, and crossover formation is eliminated or severely compromised (33, 40), demonstrating a crucial role of COSA-1/CNTD1 in converting early recombination intermediates into crossovers. Because both COSA-1 and CNTD1 are members of the cyclin family, it is plausible that they form a complex with a cyclin-dependent kinase (CDK) and regulate the recombination process through phosphorylation.

Several lines of evidence have suggested that CDK2 might be a relevant kinase partner for CNTD1. CDK2 interacts with CNTD1 in yeast-two hybrid assays (41, 42) and localizes to interstitial chromosome sites (43, 44) in a CNTD1-dependent manner (40). Reduced CDK2 activity leads to a failure in crossover formation, while a hyperactive form of CDK2 causes an increased number of MLH1 foci (45). However, due to its requirement at telomeres in tethering chromosomes to the nuclear envelope, deletion of CDK2 leads to severe defects in SC assembly between paired homologs (synapsis) and pachytene arrest (46–49). Further, while a full-length CNTD1-specific protein of the excepted size was detected (using CNTD1 antibodies) in one study (42), a short CNTD1 isoform that cannot interact with CDK2 was the predominant isoform detected in another study (using hemagglutinin antibodies in *Cntd1^FH/FH^* mice with an epitope tag sequence inserted into the endogenous *Cntd1* locus) (41), raising questions regarding the extent to which CDK2 and CNTD1 might act as functional partners. Thus, it has been difficult to determine the role of CDK2 in crossover recombination. Moreover, key meiotic targets of CDK2 have not yet been identified.

We reasoned that CDK-2, the *C. elegans* homolog of CDK2, might also localize and function at crossover sites. However, global knockdown of *C. elegans* CDK-2 by RNA interference leads to cell cycle arrest of mitotically proliferating germ cells (50), thereby precluding the analysis of its requirement during meiotic prophase. To overcome this limitation and establish the meiotic function of CDK-2, we use the auxin-inducible degradation system to deplete CDK-2 from the adult germline, demonstrating that CDK-2 partners with COSA-1 to promote crossover formation during *C. elegans* meiosis. Moreover, we identify MSH-5 as a key substrate for CDK-2 and provide evidence that CDK-2 and COSA-1 partner to promote crossover designation through phosphorylation and activation of the MutSγ complex.

## Results

### CDK-2 Colocalizes with COSA-1 Both at Early Recombination Intermediates and at Crossover Sites in Pachytene.

**Fig. 1.**
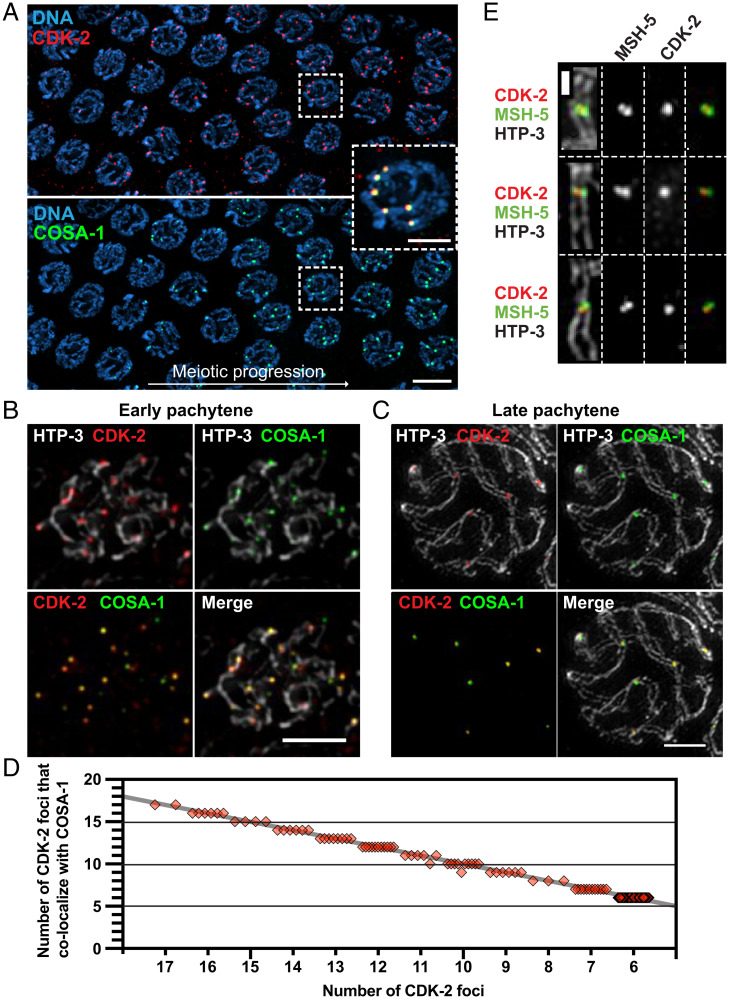
CDK-2 colocalizes with COSA-1 both at early recombination intermediates and at late pachytene crossovers. (*A*) Immunofluorescence images of a whole-mount gonad from a worm strain that expresses CDK-2::AID::3×Flag and GFP::COSA-1. (Scale bar, 5 μm.) *Inset*, CDK-2 and COSA-1 colocalizing in six bright foci in nuclei following transition to late pachytene. (Scale bar, 2 μm.) (*B* and *C*) Full projections of SIM images of spread gonads showing the staining for HTP-3 (white), CDK-2 (red), and COSA-1 (green) from early pachytene (*B*) and late pachytene nuclei (*C*). (Scale bars, 2 μm.) (*D*) Quantification of CDK-2 foci in pachytene nuclei and their colocalization with COSA-1. Each diamond represents a nucleus; the gray line indicates perfect colocalization. (*E*) Representative SIM images of individual crossover–designated sites showing CDK-2 singlet foci localizing together with MSH-5 doublets. (Scale bar, 500 nm.)

To establish the localization and function of CDK-2 during meiosis, we used CRISPR-mediated genome editing to modify the endogenous *cdk-2* locus to express CDK-2 fused to an auxin-inducible degron (AID) and three tandem Flag epitopes (3×Flag). Self-progeny of this worm strain are fully viable (100% egg viability), indicating that the AID tag does not interfere with essential CDK-2 functions. Immunofluorescence in whole-mount adult hermaphrodite germlines (XX) revealed that CDK-2 localizes to six distinct foci per nucleus in late pachytene, which correspond to crossover–designated recombination sites as marked by COSA-1 (Fig. 1*A* and *SI Appendix*, Fig. S1*A*). In male germlines (XO), CDK-2 appears as five foci in pachytene nuclei (*SI Appendix*, Fig. S1*B*), consistent with its localization to crossover sites on the five autosomes.

We used nuclear spreading and three-dimensional structured illumination microscopy (3D-SIM) to examine the localization of CDK-2 in relation to an axis component, HTP-3, and other crossover factors during meiotic progression. This cytological approach shows COSA-1 localizing to numerous early recombination intermediates as faint foci prior to transition to late pachytene (32). We likewise detected 7 to 17 CDK-2 foci in early pachytene nuclei, colocalizing with COSA-1 (Fig. 1 *B* and *D*). Upon transition to late pachytene, CDK-2 and COSA-1 are lost from most recombination sites and enriched together at six crossover–designated sites (Fig. 1 *C* and *D*). Recent evidence has further shown that a distinct substructure emerges at the crossover site, in which MSH-5 doublets appear orthogonal to chromosome axes, flanking a central COSA-1 focus (33). CDK-2 was similarly detected as a single focus positioned between two MSH-5 foci at the crossover site (Fig. 1*E*). Thus, CDK-2 colocalizes with COSA-1 at early recombination intermediates as well as at crossover–designated sites in both hermaphrodite and male germlines.

### CDK-2 Is Required for Crossover Formation.

**Fig. 2.**
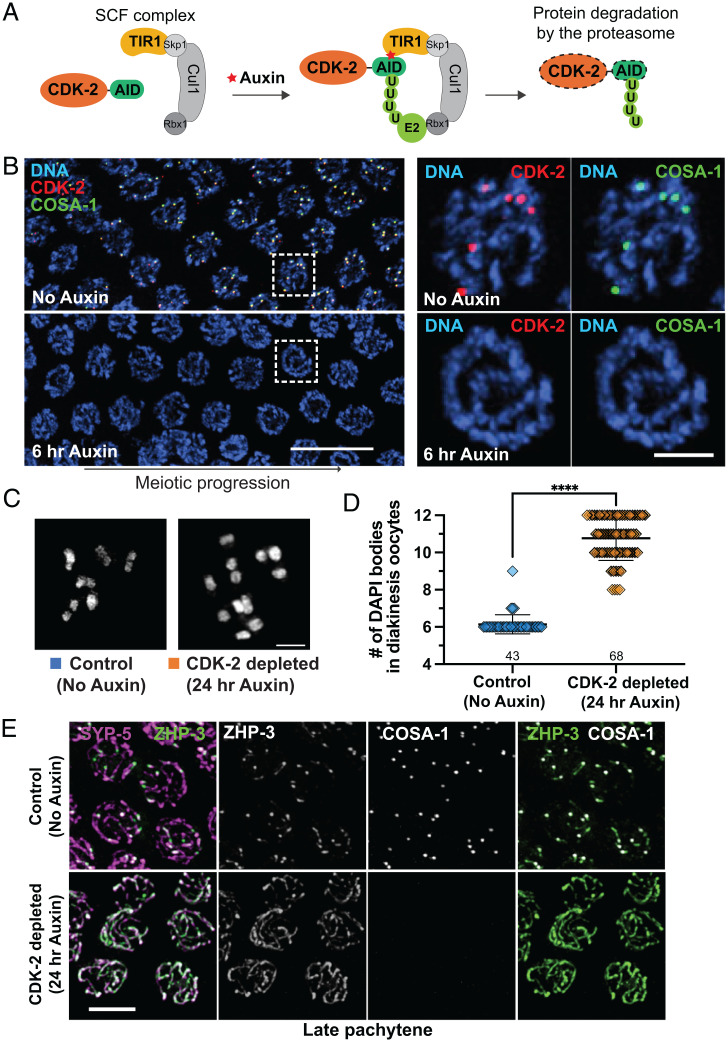
CDK-2 is required for crossover formation. (*A*) Schematic showing the auxin-inducible degradation system for CDK-2. TIR1::mRuby expressed from the *sun-1* promoter (*p_sun-1_*) enables auxin-regulated control of CDK-2 degradation specifically in the germline. (*B*) *Left*, young-adult hermaphrodites expressing CDK-2::AID::3×Flag, GFP::COSA-1, and *P_sun-1_*::TIR1::mRuby were treated with 1 mM auxin for indicated times. Immunofluorescence images of late pachytene nuclei are shown. (Scale bar, 10 μm.) *Right*, pachytene nuclei from the boxed regions at the indicated time points after 1 mM auxin treatment. (Scale bar, 2 μm.) (*C*) DAPI-stained oocyte nuclei in diakinesis from control (no auxin) and CDK-2–depleted animals (24 h auxin) fixed and stained at 24 h after L4. (Scale bar, 3 μm.) (*D*) Graph showing the number of DAPI bodies at diakinesis. Note that this assay tends to underestimate reductions in chiasma formation, as some univalents may be too close together to be resolved unambiguously; ****P < 0.0001 by Mann–Whitney *U* test. (*E*) Young-adult hermaphrodites expressing CDK-2::AID::3×Flag, GFP::COSA-1, ZHP-3::V5, and TIR1::mRuby were treated with or without 1 mM auxin for 24 h after L4 and dissected for immunofluorescence. Late pachytene nuclei stained for ZHP-3::V5 (green), SYP-5 (magenta), and GFP::COSA-1 (white) are shown. (Scale bar, 4 μm.)

To assess CDK-2 function during meiosis, we generated a strain in which CDK-2::AID::3×Flag is expressed in conjunction with germline-expressed plant F-box protein TIR1, which forms an SCF (Skp1-Cul1-F-box) E3 ubiquitin ligase to target AID-tagged proteins for degradation in the presence of auxin (51) (Fig. 2*A*). Within 6 h of 1 mM auxin treatment, CDK-2 was no longer detected in pachytene nuclei by immunofluorescence, demonstrating its rapid and efficient degradation (Fig. 2*B*). The signal for COSA-1 was also completely lost in CDK-2–depleted germlines, indicating that COSA-1 localizes to recombination sites in a CDK-2–dependent manner. Likewise, CDK-2 was not detected in pachytene nuclei of animals homozygous for a null mutation of *cosa-1* (*SI Appendix*, Fig. S2*A*), demonstrating their mutual dependence.

Depletion of CDK-2 resulted in loss of crossover–based connections (chiasmata) that maintain associations between homologs in oocytes at diakinesis, the last stage of meiotic prophase. Six DAPI-stained bodies corresponding to six pairs of homologs connected by chiasmata (bivalents) are observed in wild-type diakinesis oocytes. However, following 24-h auxin treatment, CDK-2–depleted oocytes displayed 8 to 12 DAPI-stained bodies, reflecting failure to form crossovers (Fig. 2 *C* and *D*). Further, the RING domain–containing protein ZHP-3, which normally becomes restricted to six crossover sites in late pachytene nuclei (12), failed to become restricted to foci in CDK-2–depleted gonads and instead persisted along the SC (Fig. 2*E*), reflecting a requirement for CDK-2 in crossover formation.

**Fig. 3.**
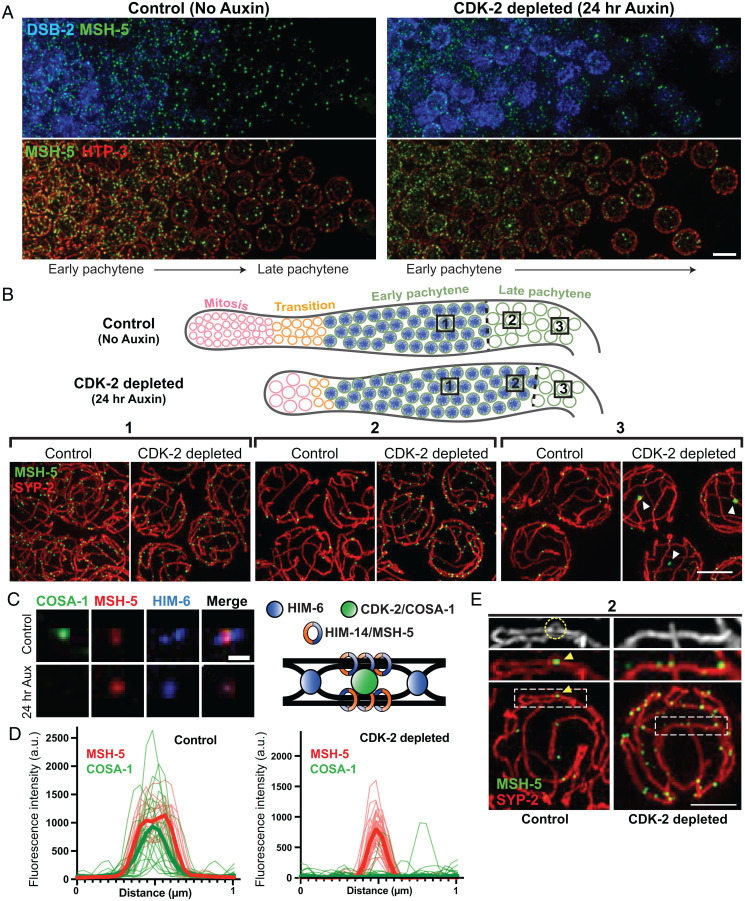
CDK-2 is required for stabilizing crossover–specific recombination intermediates. (*A*) Animals expressing CDK-2::AID::3×Flag and TIR1::mRuby were treated with or without 1 mM auxin for 24 h after L4. Dissected gonads were spread and stained for DSB-2 (blue), MSH-5 (green), and HTP-3 (red). (Scale bar, 5 μm.) (*B*) *Top*, diagram illustrating the effect of CDK-2 depletion on meiotic progression. The DSB-2–positive nuclei (shown in blue) represent nuclei in early pachytene. *Bottom*, representative SIM images of nuclei from the indicated regions (1, 2, or 3) of spread gonads from control and CDK-2–depleted worms as indicated in the diagram above. White arrowheads in 3 indicate large MSH-5 aggregates. (Scale bar, 3 μm.) (*C*) Representative fluorescent images of recombination sites in late pachytene from control versus CDK-2–depleted germline. Stainings for MSH-5, HIM-6, and COSA-1 are shown. (Scale bar, 400 nm.) A schematic depicting the hypothesized architecture of recombination factors at the crossover–designated site is shown on the right. (*D*) Line scan profiles of MSH-5 (red) and COSA-1 signals (green) at recombination sites in late pachytene nuclei from control (*n* = 26) and CDK-2–depleted germlines (*n* = 26). Thin lines are individual traces, and thick lines are averages; a.u., arbitrary units. (*E*) Representative SIM images of spread gonads from region 2 in the diagram above and a segment of SC stretch from control and CDK-2–depleted animals. MSH-5 (green) and SYP-2 (red) stainings are shown. The yellow circle and arrowhead in the control indicate the SC bubble at the crossover site. (Scale bar, 2 μm.)

Consistent with previous studies implicating *C. elegans* CDK-2 in the mitosis-to-meiosis decision and in promoting the proliferative fate of germline stem cells (50, 52), depletion of CDK-2 by 24-h auxin treatment dramatically reduced the number of germ cells in the premeiotic region of the gonad (Fig. 3*B* and *SI Appendix*, Fig. S2 *B* and *C*). However, these CDK-2–depleted gonads exhibited normal pairing of HIM-8, a protein that binds a special region on X chromosomes known as the pairing center (53), and robust synapsis (*SI Appendix*, Fig. S2 *D* and *E*). The sole RecA recombinase in *C. elegans*, RAD-51 (54, 55), was also detected as numerous foci in both control and CDK-2–depleted gonads (*SI Appendix*, Fig. S2 *F* and *G*). Thus, meiotic DSBs are induced in CDK-2–depleted germlines but cannot be processed into crossovers in the absence of CDK-2.

### CDK-2 Is Required for Formation or Stabilization of Crossover–Specific Recombination Intermediates.

Next, we used a partial nuclear spreading protocol that maintains the temporal and spatial organization of the gonad (33, 56) to visualize the effects of CDK-2 depletion on the progression and architecture of meiotic recombination sites. To this end, we tagged MSH-5 at its C terminus with the 14-amino acid (aa)–long V5 epitope in a strain expressing CDK-2::AID::3×Flag and TIR1::mRuby and visualized MSH-5::V5 together with DSB-2 [a marker of early meiotic prophase (57)], HTP-3, and/or an SC component SYP-2. As previously demonstrated (33, 39), MSH-5 localized as numerous foci corresponding to nascent recombination sites in early pachytene nuclei and ultimately pared down to six robust crossover site foci in late pachytene in controls (Fig. 3 *A* and *B*). In CDK-2–depleted germlines, however, MSH-5 persisted longer as multiple foci, and the early pachytene region positive for DSB-2 staining or phosphorylation of pairing center proteins (pHIM-8/ZIMs) was extended (Fig. 3 *A* and *B* and *SI Appendix*, Fig. S3*A*), reflecting delayed meiotic progression due to failure to form crossover intermediates (57–59). Further, although CDK-2–depleted germ cells did eventually lose the DSB-2 signal and transition into the late pachytene stage, multiple faint MSH-5 foci persisted along chromosome axes (Fig. 3*B*), implying a failure of crossover designation in the absence of CDK-2. We note that abnormally large puncta of MSH-5 (white arrowheads) were detected in late pachytene nuclei of CDK-2–depleted germlines, which likely represent pathological aggregates of MSH-5 formed in the absence of CDK-2 (*Discussion*).

Examination of spread nuclei using 3D-SIM further revealed a failure to establish normal crossover site architecture in CDK-2–depleted germlines. Recent work has shown that crossover–designated sites display a distinct spatial organization of recombination factors (33, 60). Specifically, cohorts of MSH-5 and the Bloom helicase HIM-6 are each detected as orthogonally localized doublets. This orientation is interpreted to reflect their association with different parts of an underlying dHJ, with COSA-1 localizing at the center of the cross formed by the HIM-6 and MSH-5 doublets (33). Whereas this organization was detected in late pachytene in controls, MSH-5 and HIM-6 were not detected as doublets in CDK-2–depleted germ cells (Fig. 3 *C* and *D*), consistent with a failure to form crossover–specific intermediates. Additionally, bubble-like SC structures have been detected at crossover–designated sites and are proposed to promote crossover maturation by encapsulating and protecting crossover–designated sites from anti–crossover activities (33). While SC bubbles surrounding MSH-5 foci were detected in controls, such structures were not found in CDK-2–depleted germlines (Fig. 3*E*). Taken together, our data support that CDK-2 is required for maturation of early recombination sites into crossover–specific recombination intermediates.

### Phosphorylation of MSH-5 Depends on Both CDK-2 and COSA-1 In Vivo.

Given the requirement for CDK-2 in crossover designation, we hypothesized that CDK-2 might phosphorylate pro–crossover factors, such as the MutSγ complex and the ZHP proteins, to modulate their functions. Moreover, given the colocalization and functional interdependence of CDK-2 and COSA-1, we hypothesized that COSA-1 might partner with CDK-2 to promote target phosphorylation. Experiments in which we coexpressed recombinant CDK-2–6×His and glutathione *S*-transferase–COSA-1 in insect cells provided evidence consistent with CDK-2 and COSA-1 being able to form a complex in vitro (*SI Appendix*, Fig. S4 *A* and *B*); however, we did not pursue this in vitro approach further as both proteins were largely insoluble. Instead, we sought to identify a relevant in vivo phosphorylation target.

**Fig. 4.**
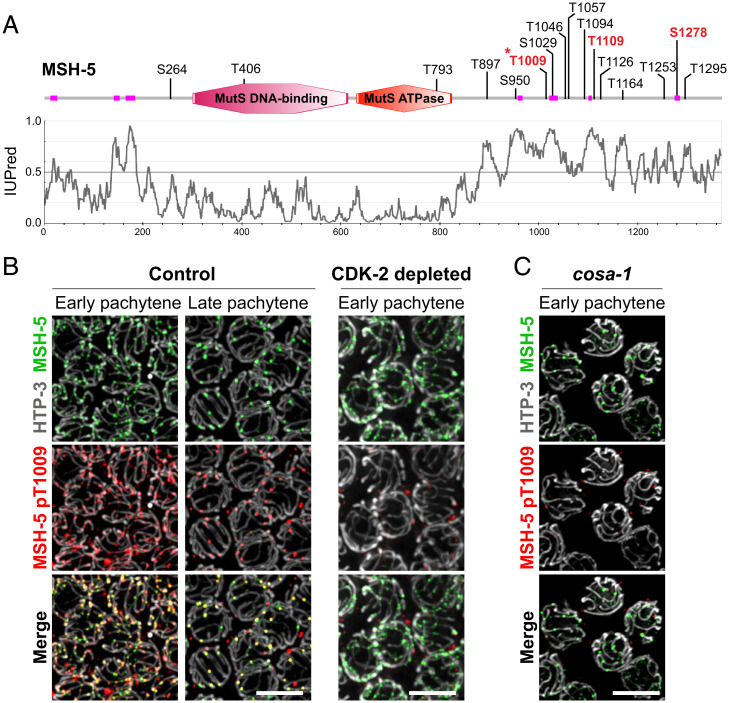
CDK-2 is responsible for MSH-5 phosphorylation within its C-terminal tail. (*A*) Schematic showing the domain structure and putative CDK phosphorylation sites in MSH-5 (adapted from the SMART database). Low-complexity regions are shown in magenta, and in vitro phosphorylation sites mapped by mass spectrometry analysis are indicated in red. An asterisk indicates the MSH-5 pT1009 epitope. The IUPred disorder score profile for MSH-5 is shown below the schematic. (*B* and *C*) Immunofluorescence images of spread pachytene nuclei from control (no auxin), CDK-2–depleted (1 mM auxin treatment for 24 h), and *cosa-1(tm3298)* germlines. (*C*) Nonspecific signals from the MSH-5 pT1009 antibody that do not colocalize with MSH-5 were occasionally detected at the nuclear periphery. Staining for HTP-3 (white), MSH-5 (green), and MSH-5 pT1009 (red) is shown. (Scale bars, 5 μm.)

We focused on MSH-5, as it contains numerous CDK consensus motifs ([S/T]P) in an extended and highly disordered C-terminal tail (Fig. 4*A*) and was previously demonstrated to be phosphorylated by human CDK1 in vitro (39). Using mass spectrometry, we mapped three sites on the MSH-5 C-terminal tail (T1009, T1109, and S1278) that are phosphorylated by human CDK1 in vitro (*SI Appendix*). We successfully generated phospho-specific antibodies against only one of these sites (MSH-5 pT1009), and we used these antibodies to demonstrate that MSH-5 is indeed phosphorylated in vivo in a CDK-2– and COSA-1–dependent manner. Immunofluorescence on spread nuclei revealed that the MSH-5 pT1009 signal was found at numerous recombination sites in early pachytene and became enriched at crossover–designated sites in late pachytene, colocalizing with MSH-5 and COSA-1 throughout meiotic progression (*SI Appendix*, Fig. S4*C* and Fig. 4*B*). Further, this phospho–MSH-5 signal is lost in worms expressing a mutant version of MSH-5 that includes a T1009A substitution (*SI Appendix*, Fig. S4*D*). Importantly, the MSH-5 pT1009 signal was abolished from recombination sites in both CDK-2–depleted and *cosa-1*–mutant gonads (Fig. 4 *B* and *C*), indicating that the MSH-5 C-terminal tail is phosphorylated in vivo in a manner dependent on both CDK-2 and COSA-1.

### The C-Terminal Tail of MSH-5 Is Essential for Accumulation of Pro–Crossover Factors at Recombination Sites.

Whereas the N-terminal 60% of the MSH-5 protein shows a high level of conservation with its orthologs throughout the eukaryotic kingdoms, the long C-terminal tail is unique to its *Caenorhabditis* orthologs (*SI Appendix*, Fig. S5*A*). Moreover, primary sequence conservation within this tail domain is very low among the *Caenorhabditis* MSH-5 orthologs, which are similar primarily in that they contain multiple (8–23) CDK consensus motifs embedded within a protein segment predicted to be highly disordered (*SI Appendix*, Fig. S5*B*). These features of the MSH-5 C-terminal tail suggest that the presence of the disordered tail and/or its ability to serve as a substrate for CDK-2 might contribute to the essential functions of MSH-5 in meiosis.

**Fig. 5.**
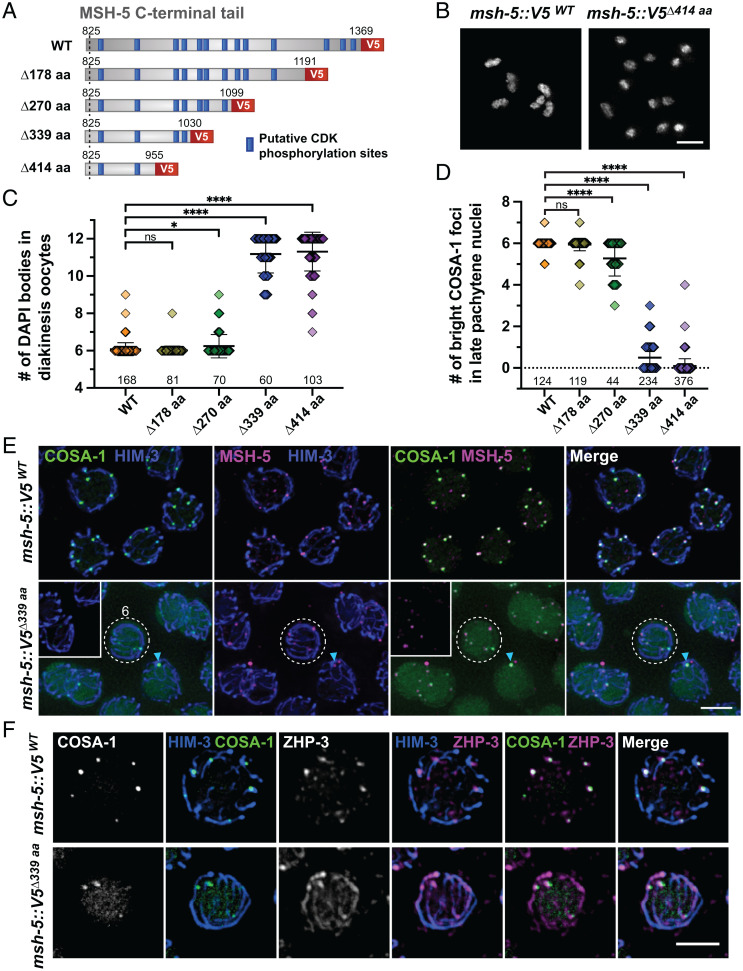
The disordered C-terminal tail of MSH-5 is essential for accumulation of pro–crossover factors at recombination sites. (*A*) Diagram of MSH-5 C-terminal truncation alleles; WT, wild type. (*B*) DAPI-stained chromosomes in diakinesis oocyte nuclei from *msh-5::V5^WT^* and *msh-5::V5^Δ414aa^* animals. (Scale bar, 3 μm.) (*C*) Quantification of the number of DAPI bodies in diakinesis oocytes from indicated genotypes. Numbers of nuclei scored are shown below the graphs. The mean ± SD is shown; ****P < 0.0001; *P = 0.0128; ns, not significant by Mann–Whitney *U* test. (*D*) Quantification of the number of bright COSA-1 foci per nucleus from indicated genotypes. Numbers of nuclei scored are shown below. The mean ± SD is shown; ****P < 0.0001; ns, not significant by Mann–Whitney *U* test. (*E*) Spread gonads from *msh-5::V5^WT^* and *msh-5::V5^Δ339aa^* animals were stained for GFP::COSA-1 (green), HIM-3 (blue), and MSH-5 (magenta). The images from *msh-5::V5^Δ339aa^* were overexposed to visualize dim COSA-1 foci; *Insets*, settings comparable to the wild-type images. Late pachytene regions are shown. A representative nucleus with six faint COSA-1 foci in the *msh-5^Δ339aa^* mutant is highlighted by a white dotted circle. A bright COSA-1 focus that colocalizes with MSH-5 in the *msh-5^Δ339aa^* is indicated by a cyan arrowhead. (Scale bar, 3 μm.) (*F*) Immunofluorescence images of spread late pachytene nuclei from *msh-5::V5^WT^* and *msh-5::V5^Δ339aa^* animals showing GFP::COSA-1 (green), HIM-3 (blue), and ZHP-3 (magenta). (Scale bar, 3 μm.)

To address this, we used CRISPR to generate worm strains expressing a series of MSH-5 C-terminal truncations by inserting V5 coding sequences followed by premature stop codons (Fig. 5*A*). Western blotting analysis showed that all four truncated proteins (Δ178 aa, Δ270 aa, Δ339 aa, and Δ414 aa) are expressed at their expected sizes, albeit at modestly reduced (50 to 70%) levels (*SI Appendix*, Fig. S6 *A* and *B*). Strikingly, truncations of MSH-5 led to a marked reduction in egg viability, and the percentage of males among surviving progeny also increased, reflecting meiotic impairment (*SI Appendix*, Fig. S6*C*). In particular, animals harboring two large truncations (*msh-5::V5^Δ339 aa^* and *msh-5::V5^Δ414 aa^*) displayed an average of ∼11 DAPI bodies in diakinesis oocytes (Fig. 5 *B* and *C* and *SI Appendix*, Fig. S6*D*) and only zero to one bright COSA-1 foci in late pachytene nuclei (Fig. 5*D* and *SI Appendix*, Fig. S6*E*). As *msh-5* is not haploinsufficient (28), these data indicate that the C-terminal tail of MSH-5 is crucial for its function in crossover formation. Immunofluorescence of spread gonads from *msh-5^Δ339aa^* mutants revealed that COSA-1 was diffusely present in the nucleoplasm (Fig. 5*E* and *SI Appendix*, Fig. S6*F*). Additionally, after spreading, dim COSA-1 foci were detected at up to six sites along chromosome axes, which colocalized with ZHP-3 and the truncated MSH-5 (Fig. 5 *E* and *F* and *SI Appendix*, Fig. S6*F*). However, ZHP-3 signal persisted along the SC in *msh-5^Δ339 aa^* mutants (Fig. 5*F*), and most recombination intermediates failed to mature into crossovers. Thus, the C-terminal tail of MSH-5 is essential for accumulation of pro–crossover factors at COSA-1–marked recombination sites.

### Phosphosites within the MSH-5 C-Terminal Tail Contribute to the Pro–Crossover Activity of the MutSγ Complex.

**Fig. 6.**
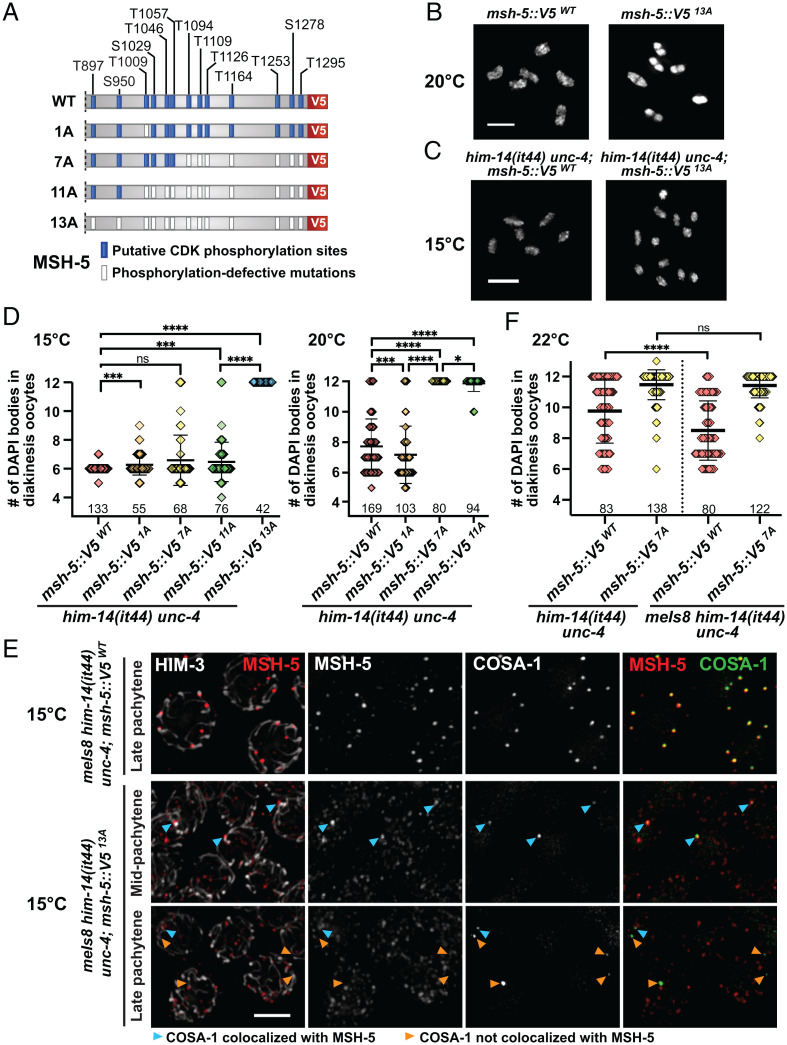
Phosphosites within the C-terminal tail of MSH-5 contributes to the pro–crossover activity of the MutSγ complex. (*A*) Diagram of *msh-5*–mutant series harboring phosphorylation-defective mutations within its C-terminal tail, indicating with white boxes the positions of S/T residues that were replaced by A residues. (*B*) Oocyte nuclei at diakinesis from *msh-5::V5^WT^* and *msh-5::V5^13A^*. Adult hermaphrodites grown at 20 °C (48 h after L4) were stained with DAPI. (Scale bar, 4 μm.) (*C*) Oocyte nuclei at diakinesis from *him-14(it44) unc-4; msh-5::V5^WT^* and *him-14(it44) unc-4; msh-5::V5^13A^*. L4 hermaphrodites were grown at 15 °C for 48 h and were dissected for DAPI staining. (Scale bar, 4 μm.) (*D*) Graph showing the number of DAPI bodies in diakinesis oocytes from indicated genotypes grown at 15 °C (*Left*) and 20 °C (*Right*). The numbers of nuclei scored are shown below. The mean ± SD is shown; ****P < 0.0001; ***P = 0.0004 to 0.0009; *P = 0.0158; ns, not significant by Mann–Whitney *U* test. (*E*) Immunofluorescence images of spread pachytene nuclei from indicated genotypes grown at 15 °C showing staining for HIM-3 (white), MSH-5::V5 (red), and GFP::COSA-1 (green). Cyan arrowheads indicate COSA-1 foci that colocalize with MSH-5, while orange arrowheads indicate the ones that do not overlap with MSH-5. (Scale bar, 3 μm.) (*F*) Graph showing the number of DAPI bodies in diakinesis oocytes from indicated genotypes grown at 22 °C. Numbers of nuclei scored are shown below. The mean ± SD is shown; ****P < 0.0001; ns, not significant by Mann–Whitney *U* test.

To determine the significance of phosphorylation of the MSH-5 tail, we sequentially mutated the codons corresponding to the 13 predicted and/or mapped CDK phosphorylation sites in its C terminus using CRISPR (Fig. 6*A* and *SI Appendix*, Fig. S7*A*). Contrary to our initial expectations based on the truncation mutants, animals homozygous for *msh*-5 phosphomutations (4A, 11A, and 13A) did not show obvious phenotypes in meiosis, exhibiting nearly normal progeny viability (*SI Appendix*, Fig. S7*B*). Even the *msh-5::V5^13A^* mutant, in which all 13 CDK consensus sites were mutated to alanines, was able to designate crossovers and form bivalents as in wild type (*SI Appendix*, Fig. S7 *C–E* and Fig. 6*B*).

As the conserved presence of multiple CDK phosphorylation sites suggests a functional importance, we hypothesized that preventing phosphorylation of the MSH-5 C terminus did not cause overt phenotypes because phosphorylation is only one of multiple parallel pathways that converge to ensure a robust outcome of meiosis. Thus, we used a temperature-sensitive allele of *him-14*/*msh-4*, which encodes the heterodimeric partner of MSH-5 in the MutSγ complex, as a sensitized genetic background to reveal a functional deficit for *msh-5* phosphomutants. *him-14(it44)* harbors a missense mutation (D406N) within its conserved DNA-binding domain and is characterized by a temperature-sensitive reduction in crossover formation (24, 61). Analysis of phosphosite mutations in this sensitized context provided clear evidence that phosphorylation of the MSH-5 tail functions to augment the pro–crossover activity of MutSγ in vivo.

First, we found that the *msh-5::V5^13A^* mutation showed strong synthetic phenotypes with *him-14(it44)* at 15 °C, a temperature that is normally permissive for *him-14(it44)* (Fig. 6 *C* and *D*). Whereas DAPI staining of diakinesis oocytes did not reveal meiotic defects in *him-14(it44); msh-5::V5^WT^* control animals at 15 °C, 12 univalents were consistently observed in *him-14(it44); msh-5::V5^13A^* oocytes, indicating a failure of crossover formation. Further, nuclear spreading and immunofluorescence revealed that while wild-type MSH-5 pared down to colocalize with six bright COSA-1 foci at crossover–designated sites in *him-14(it44)* at 15 °C, the MSH-5^13A^ protein persisted as numerous foci throughout pachytene in the *him-14(it44); msh-5::V5^13A^* double mutant (Fig. 6*E*), similar to the phenotypes observed in CDK-2–depleted germlines (Fig. 3). Moreover, in *him-14(it44); msh-5::V5^13A^* animals, one to three COSA-1 foci initially associated with MSH-5, but this colocalization was lost in late pachytene (only 33% of COSA-1 signals colocalized with MSH-5 in *him-14(it44); msh-5::V5^13A^* versus 98% in *him-14(it44); msh-5::V5^WT^*; Fig. 6*E* and *SI Appendix*, Fig. S8*A*). Interestingly, ZHP-3 was detected together with COSA-1 in late pachytene nuclei of *him-14(it44); msh-5::V5^13A^* (*SI Appendix*, Fig. S8*B*), suggesting that phosphorylation of the MSH-5 C terminus is required for retaining the association of MutSγ with other pro–crossover factors in *him-14(it44)* animals.

Second, analysis of *him-14(it44)*–double mutant animals carrying phospho-null mutations at 1, 7, or 11 phosphosites (1A, 7A, and 11A) further revealed that multiple phosphorylation sites in the MSH-5 tail can contribute to promoting MutSγ activity (Fig. 6 *A* and *D* and *SI Appendix*, Fig. S8*C*). The *him-14(it44); msh-5::V5^11A^* mutant was particularly informative, as analysis of diakinesis oocytes revealed only mild meiotic impairment at 15 °C, in striking contrast to the complete failure of crossover formation observed in *him-14(it44); msh-5::V5^13A^* oocytes under the same conditions. This suggests that phosphorylation of as few as two sites within the MSH-5 tail can sustain sufficient pro–crossover activity of the partially compromised MutSγ complexes. However, severe impairment of crossover formation is observed in both *him-14(it44); msh-5::V5^11A^* and *him-14(it44); msh-5::V5^7A^* when MutSγ function is further compromised at the semipermissive temperature of 20 °C (Fig. 6*D*, Right). This suggests that phosphorylation at additional sites can contribute to augmentation of MutSγ activity.

### MSH-5 Phosphosites Are Required for Suppression of the *him-14(it44)* Crossover Deficit by Elevated COSA-1 Levels.

The *meIs8* transgene expressing green fluorescent protein (GFP)::COSA-1 can partially alleviate the crossover deficit observed at semipermissive temperatures in the *him-14(it44)* mutant (62). Using qRT-PCR, we determined that *cosa-1* mRNA levels are up-regulated by twofold in *mels8* worms compared to wild-type controls (*SI Appendix*, Fig. S8*D*). Thus, we hypothesized that this suppression might reflect elevated activity of a putative CDK-2/COSA-1 complex, which compensates for the impaired MutSγ activity in *him-14(it44)* through hyperphosphorylation of downstream targets, including MSH-5. To test whether suppression of *him-14(it44)* by *meIs8* was dependent on phosphosites in the MSH-5 C-terminal tail, we compared the effect of *meIs8* in *him-14(it44); msh-5::V5^WT^* and *him-14(it44); msh-5::V5^7A^* genetic backgrounds, reasoning that reducing the number of available phosphosites might abrogate suppression. Experiments were conducted at 22 °C, a semipermissive temperature at which the suppression of *him-14(it44)* by *meIs8* is evident. At 22 °C, *him-14(it44); msh-5::V5^WT^* diakinesis oocytes displayed an average of 9.8 DAPI bodies, reflecting a mixture of bivalents and univalents resulting from a partial impairment of crossover formation, but the number of DAPI bodies was reduced to 8.5 in the presence of the *meIs8* transgene (Fig. 6*F* and *SI Appendix*, Fig. S8*E*), indicating an increase in bivalent formation reflecting increased success in crossover formation. However, there was no significant difference in the number of DAPI bodies, with or without the *meIs8* transgene, in the *him-14(it44); msh-5::V5^7A^* background (Fig. 6*F* and *SI Appendix*, Fig. S8*E*). This finding strongly suggests that phosphorylation of the MSH-5 C-terminal tail is required for suppression of *him-14(it44)* phenotypes by elevated CDK-2 activity, supporting the conclusion that the MSH-5 tail is the major CDK-2 target whose phosphorylation is responsible for augmenting the residual MutSγ activity in *him-14(it44)* animals.

## Discussion

### *C. elegans* CDK-2 Partners with COSA-1 to Promote Crossover Formation.

Here, we demonstrate that *C. elegans* CDK-2 localizes to recombination intermediates and partners with COSA-1 to promote crossover formation. Using superresolution microscopy, we show that CDK-2 and COSA-1 colocalize together within the same subcompartment at crossover sites, with a spatial positioning distinct from those exhibited by other recombination factors, such as MutSγ and Bloom helicase. Further, we show that CDK-2 and COSA-1 are interdependent for localization, are both required for in vivo phosphorylation of MutSγ, and their absence/depletion has identical consequences for the progression of recombination. Based on these collective findings and conservation of the predicted cyclin/CDK interface (39), the most parsimonious explanation is that CDK-2 and COSA-1 combine to form a dedicated meiotic CDK/cyclin complex that functions to convert a subset of meiotic DSBs into interhomolog crossovers by stabilizing crossover–specific recombination intermediates.

While our data strongly support *C. elegans* CDK-2 and COSA-1 working together as a functional unit in promoting meiotic crossover formation, it remains unresolved whether this functional partnership is conserved in mammalian spermatocyte meiosis. On the one hand, mouse CNTD1 and CDK2 colocalize at late crossover sites (41, 42), and loss of *Cntd1* function (40, 42), a *Cntd1^Q^* allele that disrupts a Y2H interaction between CNTD1 and CDK2 (42), and a partial loss-of-function *Cdk2^T160A^* allele (45) all cause very similar phenotypes, including elevated/persistent MSH4 foci, loss of late crossover markers, and a deficit of chiasmata. While these findings support a functional partnership, however, no immunoprecipitation studies to date have detected in vivo association between CNTD1 and CDK2. Further, detection of a short isoform of CNTD1 lacking a domain essential for cyclin/CDK interaction has led to the proposal that CNTD1 might function through alternative binding partners independently of CDK (41).

### Evidence That CDK-2 Promotes Crossover Designation through Phosphorylation of MSH-5.

**Fig. 7.**
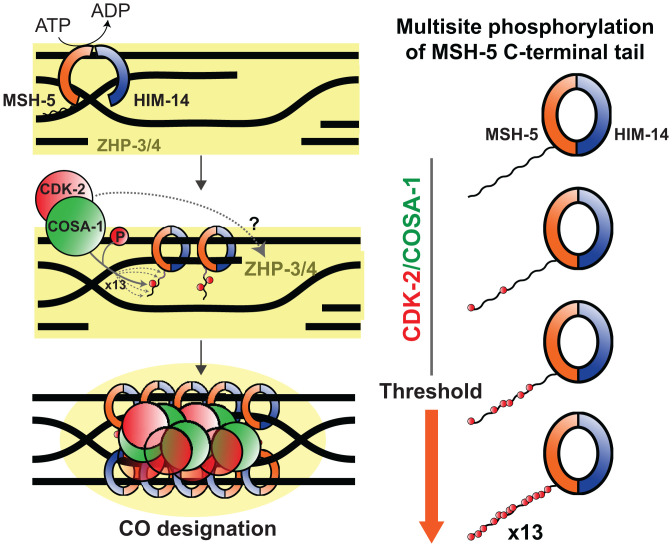
Model for robust designation of meiotic crossover sites through CDK-2/COSA-1–dependent multisite phosphorylation of the MutSγ complex, positive feedback, and scaffold-like properties of the MSH-5 tail. The MutSγ complex recognizes and binds nascent recombination intermediates. CDK-2/COSA-1 is also recruited to the early recombination sites and phosphorylates MSH-5 in its disordered C-terminal tail. As the recombination intermediate matures and recruits more MutSγ and CDK-2/COSA-1, more sites in the MSH-5 tail are phosphorylated. CDK-2/COSA-1 likely phosphorylates other substrates essential for crossover formation. Multisite phosphorylation of the MSH-5 C-terminal tail generates an ultrasensitive response that potentiates the overall activity of MutSγ to stabilize crossover–specific recombination intermediates. Phosphorylation within the MSH-5 tail also helps retain CDK-2/COSA-1 and ZHP-3/4 (depicted in yellow), thereby providing positive feedback and conferring a switch-like behavior that contributes to the robustness of crossover (CO) designation.

Here, we identify MutSγ as a key meiotic target of CDK-2 and demonstrate that MSH-5 is phosphorylated in a COSA-1– and CDK-2–dependent manner within its disordered C-terminal tail. As mutating all 13 C-terminal CDK motifs in MSH-5 does not alone result in meiotic defects, CDK-2 likely has additional substrates essential for crossover formation. However, severe consequences of phosphosite loss are evident when the activity of MutSγ is compromised, indicating the importance of phosphorylation within the MSH-5 tail for enabling success of meiosis under suboptimal conditions. The significance of this kinase–substrate relationship is further supported by dosage suppression, in which extra copies of the *cosa-1* gene enable *him-14(it44)* animals to form higher levels of crossovers at a semipermissive temperature (62). We have shown that suppression of *him-14(it44)* phenotypes by COSA-1 overexpression requires MSH-5 phosphorylation within its C-terminal region. Thus, we propose that MSH-5 is a key substrate of CDK-2/COSA-1 and that phosphorylation within the MSH-5 C-terminal tail potentiates the overall activity of MutSγ in stabilizing crossover–specific recombination intermediates (Fig. 7).

### The C-Terminal Tail of MSH-5 as a Scaffold to Accumulate Other Pro–Crossover Factors.

CDK phosphorylation sites in MSH-5 are clustered within its disordered C-terminal domain, which we have shown to be essential for crossover formation. Because the MSH-5 C-terminal tail is outside of its enzymatic core or the dimerization interface mapped for human MSH4 and MSH5 (36), it is unlikely that deleting the C-terminal tail affects the ATP hydrolysis rate or the formation of the HIM-14/MSH-5 heterodimer. In worms expressing a truncated MSH-5 (*msh-5^Δ339 aa^*), MSH-5 localizes to no more than six COSA-1–marked recombination sites, suggesting that crossover site designation may have occurred. However, pro–crossover factors do not accumulate to wild-type levels at these sites, depletion of ZHP-3 from along the length of the SC does not occur, and lack of chiasmata connecting homologs at diakinesis indicates a failure to process these recombination intermediates into crossovers (Fig. 5). We propose that the C-terminal tail of MSH-5 serves as a scaffold to accumulate proteins required for crossover formation. Indeed, intrinsically disordered proteins frequently contain short linear motifs that mediate interactions with diverse targets and have emerged as major hubs in cellular signaling (63). Recent work in mice has identified a novel proline-rich protein, PRR19, that functions with CNTD1 to promote crossover formation (42). Intriguingly, the MSH-5 tail is also enriched in prolines (CDK is a proline-directed kinase) and thus may act as a functional substitute for PRR19 to stably recruit CDK-2/COSA-1 in *C. elegans*. Determining how the MSH-5 tail mediates higher-order assemblies of pro–crossover factors will be an important topic for future research.

The phenotype observed in *msh-5^Δ339 aa^* animals is in sharp contrast to that in *msh-5::V5^13A^; him-14(it44)*, where one to three bright foci of COSA-1 initially localize to a subset of MutSγ-positive recombination intermediates but lose their association in late pachytene (Fig. 6*E*). Thus, although the MSH-5 C-terminal tail itself is required for concentrating pro–crossover factors at recombination sites, it appears to have deleterious effects on their retention in its unphosphorylated form. We speculate that the C-terminal tail of MSH-5 may also act as a scaffold for recruiting antirecombinases, which is counteracted by CDK-2–dependent phosphorylation. Recent evidence in *Saccharomyces cerevisiae* has demonstrated that phosphorylation of the N-terminal degron within Msh4 protects it from proteolysis at recombination sites, thereby activating its pro–crossover activity (64). However, phosphorylation of the MSH-5 C-terminal tail does not seem to have similar stabilizing effects, as we did not observe an increased level of MSH-5 in our C-terminal truncation mutants. As ZHP-3 is depleted from the SC in *msh-5::V5^13A^; him-14(it44)* mutants while colocalizing with COSA-1, CDK-2/COSA-1 may also phosphorylate ZHP-3/4 and trigger their relocation from the SC to crossover–designated sites (Fig. 7).

### Multisite Phosphorylation, Positive Feedback, and Propensity for Aggregation Provide a Robust Mechanism for Crossover Designation.

CDK phosphorylation sites are often clustered in disordered regions (65), and multisite phosphorylation by CDK can set a threshold to elicit an ultrasensitive response (66, 67). Our analysis of *msh-5* phosphosite mutants revealed that crossover formation becomes highly sensitive to the number of phosphorylation sites available in the MSH-5 C-terminal tail when the activity of MutSγ is compromised by a temperature-sensitive mutation in *him-14*. At more permissive temperatures where HIM-14 is largely functional, fewer phosphosites are needed to achieve a threshold level of MutSγ activity required to ensure crossover formation. Conversely, under more restrictive conditions where HIM-14 is less functional, more phosphosites are required to achieve a threshold level of MutSγ activity. Although all MSH-5 orthologs in the *Caenorhabditis* species possess numerous CDK consensus motifs in the disordered C-terminal region, these sites are poorly conserved. Thus, we speculate that the number of phosphorylation sites, rather than their exact position, influences the pro–crossover activity of MutSγ, in line with several precedents controlled by multisite phosphorylation (68, 69). Further, as phosphorylation within the MSH-5 tail promotes the stable association of CDK-2/COSA-1 to recombination sites, it can also generate positive feedback that further enhances MSH-5 phosphorylation, thereby conferring a switch-like behavior that contributes to the robustness of crossover designation.

We further speculate that the propensity for MSH-5 to form aggregates in late pachytene germ cells, revealed upon CDK-2 depletion, may also be a feature that promotes robustness of the crossover designation mechanism. Given the intrinsically disordered protein sequence in which phosphosites are embedded, we hypothesize that the MSH-5 C-terminal tail may have a capacity to undergo phase separation that can be modulated by phosphorylation, which could potentially contribute to the formation of ellipsoidal protein structures that have long been recognized as “recombination nodules” (70). The idea that formation of biomolecular condensates may contribute to crossover designation is supported by recent modeling of cytological data from *Arabidopsis*, in which HEI10 is proposed to accumulate at few sites through diffusion-mediated coarsening at the expense of smaller foci (71). By enriching pro–crossover factors at crossover–designated intermediates while depleting them from other recombination sites, the formation of biomolecular condensates can serve as a general mechanism for controlling both crossover designation and positioning.

## Materials and Methods

### *C. elegans* Genetics, Genome Engineering, and Auxin-Mediated Depletion of CDK-2.

All strains used in this study were maintained on NGM (nematode growth medium) plates seeded with OP50-1 bacteria under standard conditions as described in ref. 72. All experiments were performed at 20 °C except where noted. All *C. elegans* strains were derived from a Bristol N2 background. *SI Appendix*, Tables S1 and S3 summarize all mutations and strains used in this study. The strains expressing CDK-2::AID::3×Flag and variants of MSH-5::V5 were generated by Cas9/CRISPR-mediated homologous recombination (73). Details of procedures, CRISPR RNAs, repair templates, and genotyping primers (*SI Appendix*, Table S2) are provided in the *SI Appendix*.

Auxin-mediated degradation of CDK-2 from the *C. elegans* germline was performed as previously described (51). Briefly, auxin plates were prepared by diluting a 400 mM auxin solution (indole-3-acetic acid in ethanol) into NGM (cooled after autoclaving) to a final concentration of 1 mM. Plates were dried at room temperature and stored at 4 °C protected from light for up to 1 wk prior to use. Plates were spread with concentrated OP50-1 bacterial cultures and incubated overnight at 37 °C. Age-matched young-adult hermaphrodites were picked and left for various hours at 20 °C prior to immunofluorescence.

### Scoring DAPI-Staining Bodies in Diakinesis Oocytes of *him-14(it44)* Animals.

All strains carrying *him-14(it44) unc-4(e120)* were maintained at 20 °C with the *mnC1* balancer. *unc-4(e120)*, closely linked to *him-14* on chromosome II, was used as a marker for easy genotyping and does not elicit meiotic phenotypes. Homozygote fourth larval (L4) stage worms (Unc, nongreen) were picked and transferred to experimental temperatures (15 °C, 20 °C, or 22 °C) for ∼44 h. To score DAPI-staining bodies in diakinesis oocytes, worms were picked on to a slide with a minimal volume of M9. Excess liquid was wicked away, and animals were fixed in 15 µL of 95% ethanol. Once dry, ethanol was reapplied, and this process was repeated a total of three times. The slides were mounted using VECTASHIELD containing DAPI (Vector Laboratories, H-1200-10) and sealed. Slides were stored at 4 °C for no longer than 4 d before imaging using a standard fluorescent microscope. DAPI bodies in the nuclei of diakinesis oocytes in the −1 to −3 positions were counted.

### Phosphopeptide Antibody Production and Affinity Purification.

A synthetic phosphopeptide (TAIHIP[pT]PIQMGEAC) corresponding to the *C. elegans* MSH-5 sequence flanking threonine 1009 was generated using standard methods. The phosphopeptide was conjugated to keyhole limpet hemacyanin and injected into rabbits (Covance). To affinity purify polyclonal MSH-5 pT1009 antibodies, immune sera was first passed through SulfoLink coupling resins (Thermo Fisher, 20401) coupled to a nonphosphopeptide (TAIHIPTPIQMGEAC). Flow-through was then bound and eluted from phosphopeptide-coupled resins. The specificity of the antibodies was verified by dot blot and immunofluorescence of worm strains carrying phosphorylation-defective mutations in *msh-5*.

### Immunofluorescence.

Immunofluorescence experiments involving whole-mount gonads and spread nuclei were conducted as in refs. 33 and 56 with modifications. The antibodies used, details of procedures, and imaging acquisition and processing are provided in the *SI Appendix*. Additional methods are described in the *SI Appendix*.

## Supplementary Material

Supplementary File

## Data Availability

All study data are included in the article and/or *SI Appendix*. Materials used in this research are available on request from A.M.V. and Y.K.

## References

[1] M. MacLennan, J. H. Crichton, C. J. Playfoot, I. R. Adams, Oocyte development, meiosis and aneuploidy. Semin. Cell Dev. Biol. 45, 68–76 (2015). 26454098 10.1016/j.semcdb.2015.10.005 PMC4828587

[2] S. Keeney, C. N. Giroux, N. Kleckner, Meiosis-specific DNA double-strand breaks are catalyzed by Spo11, a member of a widely conserved protein family. Cell 88, 375–384 (1997). 9039264 10.1016/s0092-8674(00)81876-0

[3] N. Hunter, N. Kleckner, The single-end invasion: An asymmetric intermediate at the double-strand break to double-Holliday junction transition of meiotic recombination. Cell 106, 59–70 (2001). 11461702 10.1016/s0092-8674(01)00430-5

[4] A. Schwacha, N. Kleckner, Identification of double Holliday junctions as intermediates in meiotic recombination. Cell 83, 783–791 (1995). 8521495 10.1016/0092-8674(95)90191-4

[5] J. Matos, S. C. West, Holliday junction resolution: Regulation in space and time. DNA Repair 19, 176–181 (2014). 24767945 10.1016/j.dnarep.2014.03.013 PMC4065333

[6] T. Allers, M. Lichten, Differential timing and control of noncrossover and crossover recombination during meiosis. Cell 106, 47–57 (2001). 11461701 10.1016/s0092-8674(01)00416-0

[7] M.-C. Marsolier-Kergoat, M. M. Khan, J. Schott, X. Zhu, B. Llorente, Mechanistic view and genetic control of DNA recombination during meiosis. Mol. Cell 70, 9–20 (2018). 29625041 10.1016/j.molcel.2018.02.032

[8] K. Zakharyevich, S. Tang, Y. Ma, N. Hunter, Delineation of joint molecule resolution pathways in meiosis identifies a crossover-specific resolvase. Cell 149, 334–347 (2012). 22500800 10.1016/j.cell.2012.03.023 PMC3377385

[9] S. Gray, P. E. Cohen, Control of meiotic crossovers: From double-strand break formation to designation. Annu. Rev. Genet. 50, 175–210 (2016). 27648641 10.1146/annurev-genet-120215-035111 PMC5319444

[10] A. Pyatnitskaya, V. Borde, A. De Muyt, Crossing and zipping: Molecular duties of the ZMM proteins in meiosis. Chromosoma 128, 181–198 (2019). 31236671 10.1007/s00412-019-00714-8

[11] V. Jantsch , Targeted gene knockout reveals a role in meiotic recombination for ZHP-3, a Zip3-related protein in *Caenorhabditis elegans*. Mol. Cell. Biol. 24, 7998–8006 (2004). 15340062 10.1128/MCB.24.18.7998-8006.2004 PMC515049

[12] N. Bhalla, D. J. Wynne, V. Jantsch, A. F. Dernburg, ZHP-3 acts at crossovers to couple meiotic recombination with synaptonemal complex disassembly and bivalent formation in *C. elegans*. PLoS Genet. 4, e1000235 (2008). 18949042 10.1371/journal.pgen.1000235 PMC2567099

[13] H. Nguyen, S. Labella, N. Silva, V. Jantsch, M. Zetka, *C. elegans* ZHP-4 is required at multiple distinct steps in the formation of crossovers and their transition to segregation competent chiasmata. PLoS Genet. 14, e1007776 (2018). 30379819 10.1371/journal.pgen.1007776 PMC6239344

[14] L. Zhang, S. Köhler, R. Rillo-Bohn, A. F. Dernburg, A compartmentalized signaling network mediates crossover control in meiosis. eLife 7, e30789 (2018). 29521627 10.7554/eLife.30789 PMC5906097

[15] C. M. Lake , Vilya, a component of the recombination nodule, is required for meiotic double-strand break formation in *Drosophila*. eLife 4, e08287 (2015). 26452093 10.7554/eLife.08287 PMC4703084

[16] C. M. Lake , Narya, a RING finger domain-containing protein, is required for meiotic DNA double-strand break formation and crossover maturation in *Drosophila melanogaster*. PLoS Genet. 15, e1007886 (2019). 30615609 10.1371/journal.pgen.1007886 PMC6336347

[17] L. Chelysheva , The *Arabidopsis* HEI10 is a new ZMM protein related to Zip3. PLoS Genet. 8, e1002799 (2012). 22844245 10.1371/journal.pgen.1002799 PMC3405992

[18] J. O. Ward , Mutation in mouse *Hei10*, an E3 ubiquitin ligase, disrupts meiotic crossing over. PLoS Genet. 3, e139 (2007). 17784788 10.1371/journal.pgen.0030139 PMC1959360

[19] A. Reynolds , RNF212 is a dosage-sensitive regulator of crossing-over during mammalian meiosis. Nat. Genet. 45, 269–278 (2013). 23396135 10.1038/ng.2541 PMC4245152

[20] S. Agarwal, G. S. Roeder, Zip3 provides a link between recombination enzymes and synaptonemal complex proteins. Cell 102, 245–255 (2000). 10943844 10.1016/s0092-8674(00)00029-5

[21] H. B. Rao , A SUMO-ubiquitin relay recruits proteasomes to chromosome axes to regulate meiotic recombination. Science 355, 403–407 (2017). 28059716 10.1126/science.aaf6407 PMC5569317

[22] H. Qiao , Antagonistic roles of ubiquitin ligase HEI10 and SUMO ligase RNF212 regulate meiotic recombination. Nat. Genet. 46, 194–199 (2014). 24390283 10.1038/ng.2858 PMC4356240

[23] N. R. Bhagwat , SUMO is a pervasive regulator of meiosis. eLife 10, e57720 (2021). 33502312 10.7554/eLife.57720 PMC7924959

[24] J. Zalevsky, A. J. MacQueen, J. B. Duffy, K. J. Kemphues, A. M. Villeneuve, Crossing over during *Caenorhabditis elegans* meiosis requires a conserved MutS-based pathway that is partially dispensable in budding yeast. Genetics 153, 1271–1283 (1999). 10545458 10.1093/genetics/153.3.1271 PMC1460811

[25] S. S. de Vries , Mouse MutS-like protein Msh5 is required for proper chromosome synapsis in male and female meiosis. Genes Dev. 13, 523–531 (1999). 10072381 10.1101/gad.13.5.523 PMC316502

[26] P. Ross-Macdonald, G. S. Roeder, Mutation of a meiosis-specific MutS homolog decreases crossing over but not mismatch correction. Cell 79, 1069–1080 (1994). 8001134 10.1016/0092-8674(94)90037-x

[27] N. M. Hollingsworth, L. Ponte, C. Halsey, MSH5, a novel MutS homolog, facilitates meiotic reciprocal recombination between homologs in *Saccharomyces cerevisiae* but not mismatch repair. Genes Dev. 9, 1728–1739 (1995). 7622037 10.1101/gad.9.14.1728

[28] K. O. Kelly, A. F. Dernburg, G. M. Stanfield, A. M. Villeneuve, *Caenorhabditis elegans* MSH-5 is required for both normal and radiation-induced meiotic crossing over but not for completion of meiosis. Genetics 156, 617–630 (2000). 11014811 10.1093/genetics/156.2.617 PMC1461284

[29] J. D. Higgins , AtMSH5 partners AtMSH4 in the class I meiotic crossover pathway in *Arabidopsis thaliana*, but is not required for synapsis. Plant J. 55, 28–39 (2008). 18318687 10.1111/j.1365-313X.2008.03470.x

[30] J. D. Higgins, S. J. Armstrong, F. C. H. Franklin, G. H. Jones, The *Arabidopsis* MutS homolog AtMSH4 functions at an early step in recombination: Evidence for two classes of recombination in *Arabidopsis*. Genes Dev. 18, 2557–2570 (2004). 15489296 10.1101/gad.317504 PMC529542

[31] C. R. Milano , Mutation of the ATPase domain of MutS homolog-5 (MSH5) reveals a requirement for a functional MutSγ complex for all crossovers in mammalian meiosis. G3 (Bethesda) 9, 1839–1850 (2019). 30944090 10.1534/g3.119.400074 PMC6553527

[32] B. Kneitz , MutS homolog 4 localization to meiotic chromosomes is required for chromosome pairing during meiosis in male and female mice. Genes Dev. 14, 1085–1097 (2000). 10809667 PMC316572

[33] A. Woglar, A. M. Villeneuve, Dynamic architecture of DNA repair complexes and the synaptonemal complex at sites of meiotic recombination. Cell 173, 1678–1691 (2018). 29754818 10.1016/j.cell.2018.03.066 PMC6003859

[34] S. Lahiri, Y. Li, M. M. Hingorani, I. Mukerji, MutSγ-induced DNA conformational changes provide insights into its role in meiotic recombination. Biophys. J. 115, 2087–2101 (2018). 30467025 10.1016/j.bpj.2018.10.029 PMC6289823

[35] T. Snowden, S. Acharya, C. Butz, M. Berardini, R. Fishel, hMSH4-hMSH5 recognizes Holliday junctions and forms a meiosis-specific sliding clamp that embraces homologous chromosomes. Mol. Cell 15, 437–451 (2004). 15304223 10.1016/j.molcel.2004.06.040

[36] T. Snowden, K.-S. Shim, C. Schmutte, S. Acharya, R. Fishel, hMSH4-hMSH5 adenosine nucleotide processing and interactions with homologous recombination machinery. J. Biol. Chem. 283, 145–154 (2008). 17977839 10.1074/jbc.M704060200 PMC2841433

[37] E. Cannavo , Regulation of the MLH1-MLH3 endonuclease in meiosis. Nature 586, 618–622 (2020). 32814904 10.1038/s41586-020-2592-2

[38] D. S. Kulkarni , PCNA activates the MutLγ endonuclease to promote meiotic crossing over. Nature 586, 623–627 (2020). 32814343 10.1038/s41586-020-2645-6 PMC8284803

[39] R. Yokoo , COSA-1 reveals robust homeostasis and separable licensing and reinforcement steps governing meiotic crossovers. Cell 149, 75–87 (2012). 22464324 10.1016/j.cell.2012.01.052 PMC3339199

[40] J. K. Holloway, X. Sun, R. Yokoo, A. M. Villeneuve, P. E. Cohen, Mammalian CNTD1 is critical for meiotic crossover maturation and deselection of excess precrossover sites. J. Cell Biol. 205, 633–641 (2014). 24891606 10.1083/jcb.201401122 PMC4050721

[41] S. Gray, E. R. Santiago, J. S. Chappie, P. E. Cohen, Cyclin N-terminal domain-containing-1 coordinates meiotic crossover formation with cell-cycle progression in a cyclin-independent manner. Cell Rep. 32, 107858 (2020). 32640224 10.1016/j.celrep.2020.107858 PMC7341696

[42] A. Bondarieva , Proline-rich protein PRR19 functions with cyclin-like CNTD1 to promote meiotic crossing over in mouse. Nat. Commun. 11, 3101 (2020). 32555348 10.1038/s41467-020-16885-3 PMC7303132

[43] T. Ashley, D. Walpita, D. G. de Rooij, Localization of two mammalian cyclin dependent kinases during mammalian meiosis. J. Cell Sci. 114, 685–693 (2001). 11171374 10.1242/jcs.114.4.685

[44] W. Liu , Phosphorylation of CDK2 at threonine 160 regulates meiotic pachytene and diplotene progression in mice. Dev. Biol. 392, 108–116 (2014). 24797635 10.1016/j.ydbio.2014.04.018

[45] N. Palmer , A novel function for CDK2 activity at meiotic crossover sites. PLoS Biol. 18, e3000903 (2020). 33075054 10.1371/journal.pbio.3000903 PMC7595640

[46] A. Viera , CDK2 is required for proper homologous pairing, recombination and sex-body formation during male mouse meiosis. J. Cell Sci. 122, 2149–2159 (2009). 19494131 10.1242/jcs.046706

[47] A. Viera , CDK2 regulates nuclear envelope protein dynamics and telomere attachment in mouse meiotic prophase. J. Cell Sci. 128, 88–99 (2015). 25380821 10.1242/jcs.154922

[48] S. Ortega , Cyclin-dependent kinase 2 is essential for meiosis but not for mitotic cell division in mice. Nat. Genet. 35, 25–31 (2003). 12923533 10.1038/ng1232

[49] C. Berthet, E. Aleem, V. Coppola, L. Tessarollo, P. Kaldis, Cdk2 knockout mice are viable. Curr. Biol. 13, 1775–1785 (2003). 14561402 10.1016/j.cub.2003.09.024

[50] J. Jeong, J. M. Verheyden, J. Kimble, Cyclin E and Cdk2 control GLD-1, the mitosis/meiosis decision, and germline stem cells in *Caenorhabditis elegans*. PLoS Genet. 7, e1001348 (2011). 21455289 10.1371/journal.pgen.1001348 PMC3063749

[51] L. Zhang, J. D. Ward, Z. Cheng, A. F. Dernburg, The auxin-inducible degradation (AID) system enables versatile conditional protein depletion in *C. elegans*. Development 142, 4374–4384 (2015). 26552885 10.1242/dev.129635 PMC4689222

[52] P. M. Fox , Cyclin E and CDK-2 regulate proliferative cell fate and cell cycle progression in the *C. elegans* germline. Development 138, 2223–2234 (2011). 21558371 10.1242/dev.059535 PMC3091494

[53] C. M. Phillips , HIM-8 binds to the X chromosome pairing center and mediates chromosome-specific meiotic synapsis. Cell 123, 1051–1063 (2005). 16360035 10.1016/j.cell.2005.09.035 PMC4435792

[54] A. Alpi, P. Pasierbek, A. Gartner, J. Loidl, Genetic and cytological characterization of the recombination protein RAD-51 in *Caenorhabditis elegans*. Chromosoma 112, 6–16 (2003). 12684824 10.1007/s00412-003-0237-5

[55] M. P. Colaiácovo , Synaptonemal complex assembly in *C. elegans* is dispensable for loading strand-exchange proteins but critical for proper completion of recombination. Dev. Cell 5, 463–474 (2003). 12967565 10.1016/s1534-5807(03)00232-6

[56] D. Pattabiraman, B. Roelens, A. Woglar, A. M. Villeneuve, Meiotic recombination modulates the structure and dynamics of the synaptonemal complex during *C. elegans* meiosis. PLoS Genet. 13, e1006670 (2017). 28339470 10.1371/journal.pgen.1006670 PMC5384771

[57] S. Rosu , The *C. elegans* DSB-2 protein reveals a regulatory network that controls competence for meiotic DSB formation and promotes crossover assurance. PLoS Genet. 9, e1003674 (2013). 23950729 10.1371/journal.pgen.1003674 PMC3738457

[58] E. L. Stamper , Identification of DSB-1, a protein required for initiation of meiotic recombination in *Caenorhabditis elegans*, illuminates a crossover assurance checkpoint. PLoS Genet. 9, e1003679 (2013). 23990794 10.1371/journal.pgen.1003679 PMC3749324

[59] Y. Kim, N. Kostow, A. F. Dernburg, The chromosome axis mediates feedback control of CHK-2 to ensure crossover formation in *C. elegans*. Dev. Cell 35, 247–261 (2015). 26506311 10.1016/j.devcel.2015.09.021 PMC4624198

[60] M. Jagut , Separable roles for a *Caenorhabditis elegans* RMI1 homolog in promoting and antagonizing meiotic crossovers ensure faithful chromosome inheritance. PLoS Biol. 14, e1002412 (2016). 27011106 10.1371/journal.pbio.1002412 PMC4807110

[61] M. C. Zetka, A. M. Rose, Mutant rec-1 eliminates the meiotic pattern of crossing over in *Caenorhabditis elegans*. Genetics 141, 1339–1349 (1995). 8601478 10.1093/genetics/141.4.1339 PMC1206871

[62] C. Girard, C. C. Akerib, A. M. Villeneuve, Suppression of *him-14(it44ts)* by a transgene insertion expressing GFP::COSA-1. MicroPub Biol., 10.17912/micropub.biology.000430 (2021). PMC8385548 34458691

[63] P. E. Wright, H. J. Dyson, Intrinsically disordered proteins in cellular signalling and regulation. Nat. Rev. Mol. Cell Biol. 16, 18–29 (2015). 25531225 10.1038/nrm3920 PMC4405151

[64] W. He , Regulated proteolysis of MutSγ controls meiotic crossing over. Mol. Cell 78, 168–183 (2020). 32130890 10.1016/j.molcel.2020.02.001 PMC7289160

[65] L. J. Holt , Global analysis of Cdk1 substrate phosphorylation sites provides insights into evolution. Science 325, 1682–1686 (2009). 19779198 10.1126/science.1172867 PMC2813701

[66] P. Nash , Multisite phosphorylation of a CDK inhibitor sets a threshold for the onset of DNA replication. Nature 414, 514–521 (2001). 11734846 10.1038/35107009

[67] J. E. Ferrell, Jr, S. H. Ha, Ultrasensitivity part II: Multisite phosphorylation, stoichiometric inhibitors, and positive feedback. Trends Biochem. Sci. 39, 556–569 (2014). 25440716 10.1016/j.tibs.2014.09.003 PMC4435807

[68] T. Doan, A. Mendez, P. B. Detwiler, J. Chen, F. Rieke, Multiple phosphorylation sites confer reproducibility of the rod’s single-photon responses. Science 313, 530–533 (2006). 16873665 10.1126/science.1126612

[69] S. C. Strickfaden , A mechanism for cell-cycle regulation of MAP kinase signaling in a yeast differentiation pathway. Cell 128, 519–531 (2007). 17289571 10.1016/j.cell.2006.12.032 PMC1847584

[70] A. T. C. Carpenter, Electron microscopy of meiosis in *Drosophila melanogaster* females: II. The recombination nodule—a recombination-associated structure at pachytene? Proc. Natl. Acad. Sci. U.S.A. 72, 3186–3189 (1975). 810799 10.1073/pnas.72.8.3186 PMC432946

[71] C. Morgan , Diffusion-mediated HEI10 coarsening can explain meiotic crossover positioning in *Arabidopsis*. Nat. Commun. 12, 4674 (2021). 34344879 10.1038/s41467-021-24827-w PMC8333306

[72] S. Brenner, The genetics of *Caenorhabditis elegans*. Genetics 77, 71–94 (1974). 4366476 10.1093/genetics/77.1.71 PMC1213120

[73] G. A. Dokshin, K. S. Ghanta, K. M. Piscopo, C. C. Mello, Robust genome editing with short single-stranded and long, partially single-stranded DNA donors in *Caenorhabditis elegans*. Genetics 210, 781–787 (2018). 30213854 10.1534/genetics.118.301532 PMC6218216

