# Supplementary material for: Robust designation of meiotic crossover sites by CDK-2 through phosphorylation of the MutSγ complex
Source: Proc Natl Acad Sci U S A. 2022 May 16;119(21):e2117865119. doi: 10.1073/pnas.2117865119 (PMC9173770; doi:10.1073/pnas.2117865119)
Supplement: Supplementary File [file pnas.2117865119.sapp.pdf]

PNAS
www.pnas.org

Supplementary Information for

Robust designation of meiotic crossover sites by CDK-2 through phosphorylation of the MutSγ complex

Jocelyn Haversat, Alexander Woglar, Kayla Klatt, Chantal C. Akerib, Victoria Roberts, Shin-Yu Chen, Swathi Arur, Anne M. Villeneuve, and Yumi Kim

*Correspondence: annev@stanford.edu and yumi.kim@jhu.edu

This PDF file includes:

Supplementary text
Figures S1 to S8
Tables S1 to S5
SI References

Supplementary Information Text

Supplementary Materials and Methods

Generation of *C. elegans* strains by CRISPR-mediated genome editing

To generate strains expressing CDK-2::AID::3xFlag and variants of MSH-5::V5, worms expressing GFP::COSA-1 and TIR1::mRuby under the *sun-1* promoter were injected with 0.25 μg/μL of Cas9 (2 nmol) complexed with 10 μM tracrRNA/crRNA oligos (IDT), 40 ng/μL of pRF4::*rol-6*(*su1006*), and a ssDNA oligo (200 ng/μL) (IDT) with 35 bp homology arms on both sides or gBlock (50 ng/μL) as a repair template (**Table S2**). F1 progeny were lysed and genotyped by PCR to detect successful CRISPR edits (**Table S2**). The correct insertion was validated by sequencing.

To mutate putative phosphorylation sites within MSH-5 C-terminal tail, serine-to-alanine or threonine-to-alanine mutations were sequentially introduced by CRISPR. The truncation mutant series of MSH-5::V5 were generated by inserting a V5 tag with a GS linker (GGATCG) followed by a premature stop codon at desired truncation sites. The correct mutagenesis was validated by sequencing, and the strains were outcrossed with N2 3 times prior to analyses.

Egg count assay

To determine egg viability, brood size, and male progeny, L4 hermaphrodites were picked onto individual plates and moved every 12 hours. Eggs were counted upon time of transfer, and surviving males and hermaphrodites were counted upon reaching adult stage.

Mapping CDK phosphorylation sites on MSH-5 *in vitro*

The full-length cDNA of *C. elegans* MSH-5 was cloned into the pTrcHis Topo Vector (Thermo Fisher K4410-01) to produce recombinant MSH-5 tagged with 6XHis at its N-terminus. Clones were verified by sequencing for orientation and sequence integrity. Protein expression was induced in BL21(DE3) cells (Sigma 69450) at 20°C using 1 mM isopropyl β-D-1-thiogalactopyranoside (dioxane free) for 16 hr. Proteins were then purified using Ni-NTA agarose (Qiagen 30210). The amount of purified protein was estimated both by conducting BCA assays (Thermo Fisher 23225) and by comparing band intensities of purified protein samples with a dilution series of 2 mg/mL Bovine Serum Albumin standard (Thermo Fisher 23209) on a Coomassie-stained SDS-PAGE gel. *In vitro* kinase reactions using purified CDK1/CyclinA2 kinase (Promega V2961) were carried out in a 25 μl reaction containing 100 ng of 6His-MSH-5, 50 μM cold ATP (Sigma), and 5U of active CDK1/CyclinA2 enzyme in 25 mM Tris pH7.0, 0.1 mM EGTA, 10 mM magnesium acetate. Reactions were terminated after 1 hour at 30°C. The protein was then purified using Phos-tag Agarose Resins (Wako AG-501) to enrich for the phosphoproteins and submitted to the TAPLIN Mass Spectrometry Facility for identification of phosphorylated peptides.

Immunofluorescence

Young adult hermaphrodites (24 hr post L4) were dissected in Egg buffer (25 mM HEPES pH 7.4, 118 mM NaCl, 48 mM KCl, 2 mM EDTA, 5 mM EGTA, 0.1% Tween-20, 15 mM NaN_3) and briefly fixed in 1% formaldehyde or for 5 minutes in 1% PFA (ZHP-3 staining shown in Figures 6 and S7B). Gonads were flash-frozen in liquid nitrogen, freeze-cracked and fixed in -20°C methanol for less than 1 minute. Fixed gonads were rehydrated in PBST (PBS and 0.1% Tween 20) and blocked with Roche Blocking reagent (Sigma 11096176001) for 35 minutes at room temperature. Samples were incubated with primary antibodies overnight at 4°C at the indicated dilutions: mouse anti-FLAG (1:200, Sigma F1804), GFP VHH (1:200, Chromotek gt-250), guinea pig anti-HTP-3 (1:500) (2), chicken anti-HIM-3 (1:500) (2), rabbit anti-phospho-HIM-8/ZIMs

(1:1000) (3), rabbit anti-MSH-5 (1:10000, SDI/Novus 38750002), chicken anti-RAD-51 (1:1000) (4), rabbit anti-MSH-5 pT1009 (1:200, this study), mouse anti-V5 (1:200, Invitrogen R960-25), rabbit anti-DSB-2 (1:5000) (5), rabbit anti-SYP-1 (1:300) (6), rabbit anti-SYP-2 (1:200) (7), rabbit anti-SYP-5 (1:500) (2), guinea-pig anti-ZHP-3 (1:100) (8), and rat HIM-8 (1:500) (9). Slides were washed with PBST and incubated with secondary antibodies for 30 minutes at room temperature at a 1:200 dilution (Sigma or Invitrogen Alexa 488, Alexa 555 or Alexa 647). Slides were washed again in PBST, stained with DAPI, and mounted in ProLong Gold (Invitrogen P36930). Slides were cured for 1-3 days and imaged using a DeltaVision^{TM} Elite system (GE) equipped with an 100x oil-immersion, 1.4 NA objective and a sCMOS camera (PCO). 3D image stacks were collected at 0.2 µm intervals and processed by iterative deconvolution (enhanced ratio, 20 cycles) and projected using the SoftWoRx package. Composite images were assembled and colored using Adobe Photoshop.

Chromosome spreading under high (Figures 1B, 1C, 3A, 3B, 3E, 4B, 4C, 5E, 5F, 6E, S4C, S4D and S6F) and low salt (Figures 1E and 3C) conditions was performed as previously described (10). Young adult hermaphrodites were dissected in 20 µl dissecting solution (0.1% v/v Tween-20, Hank's Balanced Salt Solution (Gibco 24020117) (high salt- 85% v/v, low salt- 10% v/v)) on an ethanol-washed 22x40 mm coverslip. Dissected gonads were spread across the cover slip using a pipette tip in 50 µl of spreading solution (2.5% w/v paraformaldehyde, 2% w/v sucrose, 0.32% v/v Lipsol, 0.04% w/v Sarcosyl). Coverslips were dried at room temperature overnight and washed in -20°C methanol for 20 minutes. After rehydrating in PBST (3 x 5 min), samples were processed for immunofluorescence using antibodies at the concentrations listed above.

Three-dimensional structured illumination microscopy (3D-SIM)

Samples were imaged, deconvolved and 3D-SIM reconstructed as conducted in (Pattabiraman et al., 2017). Slides were prepared by chromosome spreading (described above) and imaged as 125 nm spaced Z-stacks using a DeltaVision OMX Blaze microscopy system (GE) equipped with a 100x NA 1.40 objective. SoftWoRx was used to 3D-reconstruct images and correct for registration. Images were projected for display using maximum intensity projection in Fiji or SoftWoRx, and individual color channel contrast and brightness were adjusted for figures in Fiji.

Expression and purification of recombinant CDK-2 and COSA-1 proteins

Synthetic gene fragments (IDT) encoding the full-length open reading frames of *C. elegans* CDK-2 and COSA-1 were cloned into pFastBac1 (Gibco) **(Table S4)**. For protein purification, COSA-1 was tagged at its N-terminus with glutathione S-transferase (GST) and CDK-2 was C-terminally tagged with 6xHis. GST-COSA-1 and CDK-2-6xHis were co-expressed and purified from Sf9 cells using the Bac-to-Bac system (Life Technologies). Infected High-Five cells (Life Technologies) were lysed in 50 mM Tris pH 7.5, 20 mM imidazole, 500 mM NaCl, 0.5 mM EGTA, 1% NP-40, 1 mM DTT, 1 mM PMSF, Complete Protease inhibitor Cocktail (Roche 11873580001) by dounce homogenization followed by sonication. 6xHis-tagged CDK-2 was purified using Ni-NTA resins (Qiagen 30210) and eluted in PBS, 500 mM imidazole, and 1 mM DTT. Purified proteins were separated by SDS-PAGE and analyzed by Coomassie blue staining and immunoblots.

Immunoblots

Protein samples were separated by SDS-PAGE and transferred onto PVDF membranes (Bio-Rad) by wet transfer. Membranes were blocked in 5% milk in PBST for 1 hour at room temperature and incubated overnight at 4°C with agitation in primary antibody at the following dilutions: rabbit anti-His (1:1,000, Cell Signaling, 2365), rabbit anti-GST (1:1,000, Invitrogen, PA1-982A), mouse anti-V5 (1:1,000, Abcam, ab27671), and rabbit anti-SYP-2 (1:1000, (7)).

RNA extraction and quantitative RT-PCR

Wild-type (N2) and AV630 (*meIs8)* worms were collected in TRI Reagent (ThermoFisher, AM9738) and were subjected to three freeze-thaw cycles. Following the addition of 1-bromo-3-chloropropane, the aqueous phase was mixed with isopropanol for 2 hours at -80°C, and RNA was pelleted by centrifugation at 20,000xg for 30 minutes at 4°C. The pellet was then washed three times in 70% ethanol and resuspended in water. NanoDrop 2000 Spectrophotometer (Thermo Scientific) was used to determine the concentration and quality of RNA.

cDNA was synthesized from 500 ng of total RNA using MultiScribe Reverse Transcriptase (Invitrogen, 4311235), and *cosa-1*, *msh-5*, and *syp-2* mRNA levels were analyzed with a CFX96 Real-Time PCR System (BioRad) using ABsolute Blue qPCR SYBR Green (ThermoFisher) and the primers listed in Table S5. Two sets of primers were designed to detect the overall *cosa-1* mRNA (oYK1697 and oYK1698) vs. *cosa-1* mRNA expressed from the endogenous gene (oYK1686 and oYK1687). Relative *cosa-1* mRNA levels were calculated by the $\Delta\Delta\text{-}C_t$ method after normalization to the endogenous *cosa-1, msh-5,* and *syp-2* (11). Results presented are the average of independent calculations from biological quadruplicates.

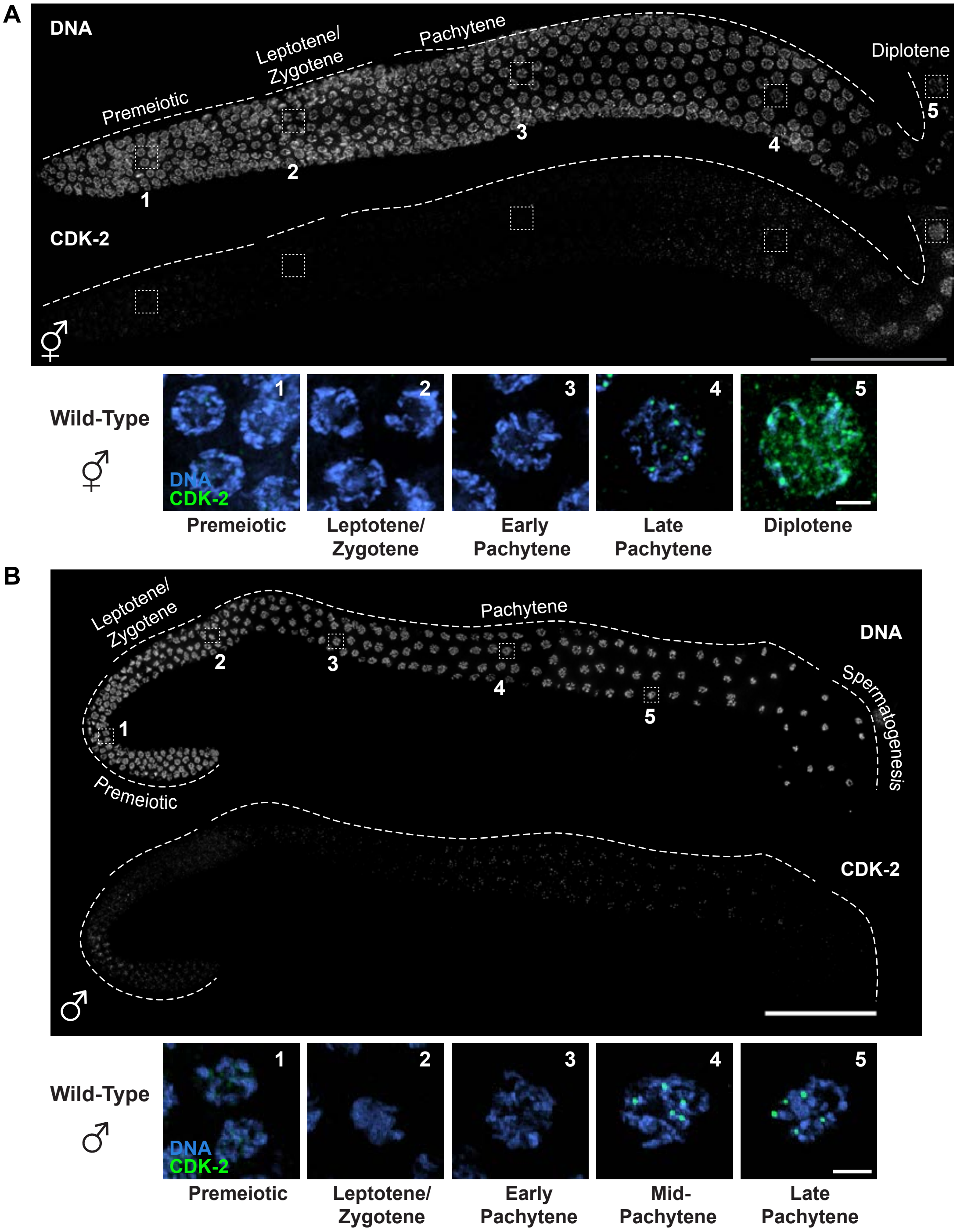

Fig. S1. CDK-2 localizes to crossover sites in both hermaphrodite and male germlines
Composite immunofluorescence images of whole mount gonads dissected from (A) an adult hermaphrodite (XX) and (B) an adult male (XO) from a strain expressing CDK-2::AID::3xFlag. Scale bars, 50 μm. Insets show representative nuclei from the boxed regions, stained for DNA (blue) and CDK-2 (green). Scale bar, 2 μm. There is no evident difference in CDK-2 localization at crossover site foci in pachytene nuclei between hermaphrodite oogenesis (6 crossovers) and male spermatogenesis (5 crossovers). The gametogenesis programs diverge substantially after completion of crossover formation; thus, the diffuse CDK-2 signal detected in the nucleoplasm of

enlarging oocytes but not in spermatocyte nuclei in the karyosome stage of spermatogenesis is not germane to the role of CDK-2 in meiotic recombination.

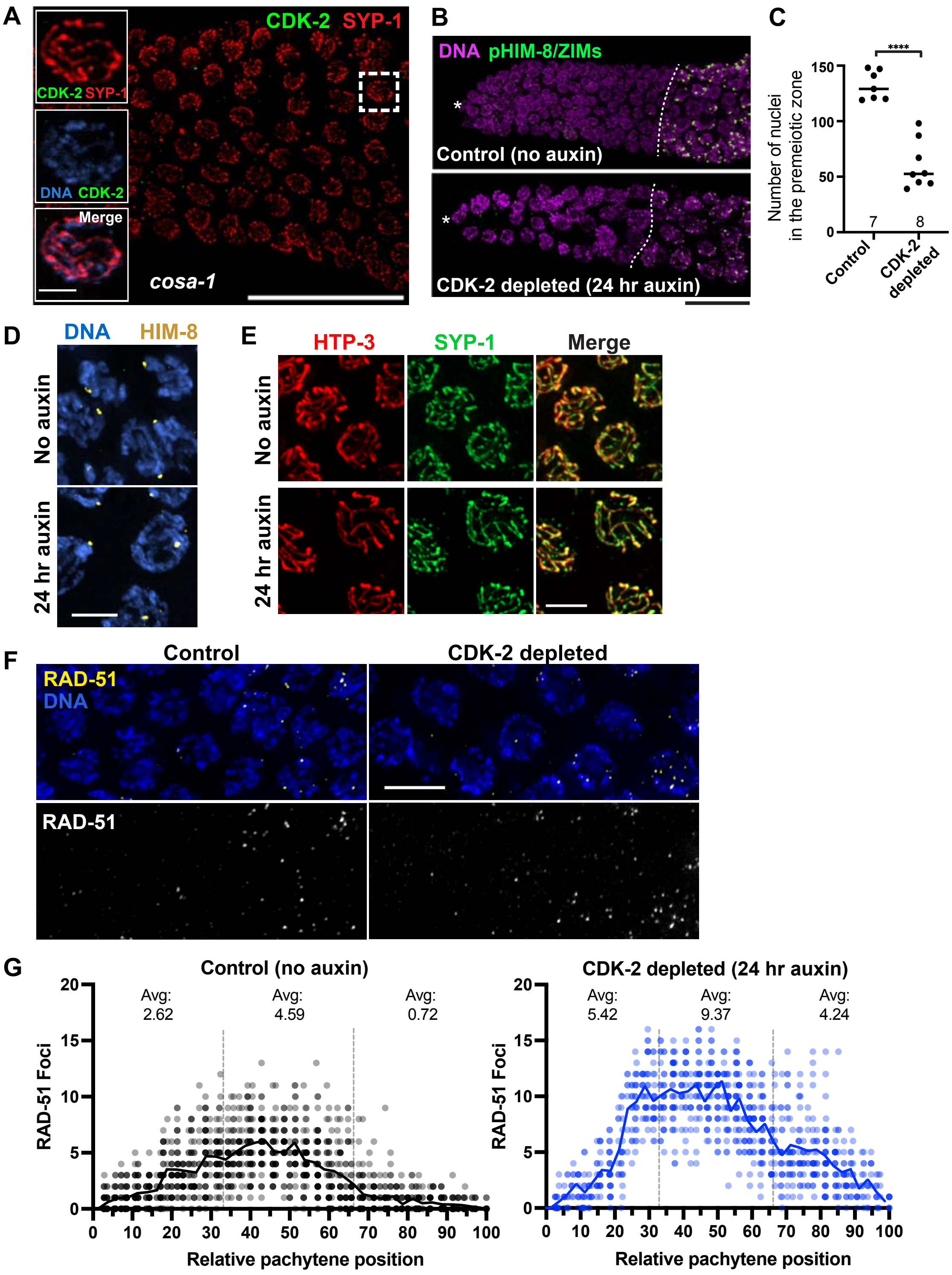

Fig. S2. CDK-2 is dispensable for homolog pairing, synapsis, and initiation of meiotic recombination

(A) Immunofluorescence images of a *cosa-1* mutant germline expressing CDK-2::AID::3xFlag showing SYP-1 (red), DNA (blue) and Flag (green). Scale bar, 25 μm; inset scale bar, 2 μm. (B) Young adult hermaphrodites expressing CDK-2::AID::3xFlag, GFP::COSA-1, and *P*$_{sun-}$

$_{1}$::TIR1::mRuby were treated with 1 mM auxin for 24 hr. Composite immunofluorescence images of distal germlines from control (no auxin) and CDK-2-depleted (24 hr auxin) animals showing DNA (magenta) and pHIM-8/ZIMs staining (green) to mark meiotic onset. Asterisks indicate the distal tip of the germline, and dotted lines indicate meiotic entry. Scale bar, 15 μm. (C) Graph showing the number of nuclei in the premeiotic zone in control (n = 7) and CDK-2-depleted germlines (n = 8). ****p<0.0001 by nonparametric unpaired t-test. (D) Immunofluorescence images of early pachytene nuclei from control (no auxin) and CDK-2-depleted (24 hr auxin) gonads stained for DNA (blue) and HIM-8 (yellow). Scale bar, 3 μm. (E) Immunofluorescence images of mid-pachytene nuclei from control (no auxin) and CDK-2-depleted (24 hr auxin) gonads stained for HTP-3 (red) and SYP-1 (green). Scale bar, 3 μm. (F) Immunofluorescence images of nuclei from early pachytene showing DNA (blue) and RAD-51 staining (yellow or white) in control (no auxin) and CDK-2-depleted (24 hr auxin) gonads. Scale bar, 5 μm. (G) Graphs showing the quantification of RAD-51 foci in the pachytene region of control (no auxin; n=3) and CDK-2-depleted (24 hr auxin; n=3) gonads. RAD-51 foci within individual nuclei were counted manually and plotted against relative pachytene position, where 0 % corresponded to the start of RAD-51 loading and 100% corresponded to the end of pachytene. RAD-51 levels are elevated, and RAD-51 foci persist longer in CDK-2 depleted germlines, consistent with increased meiotic DSBs and a delayed meiotic progression.

A

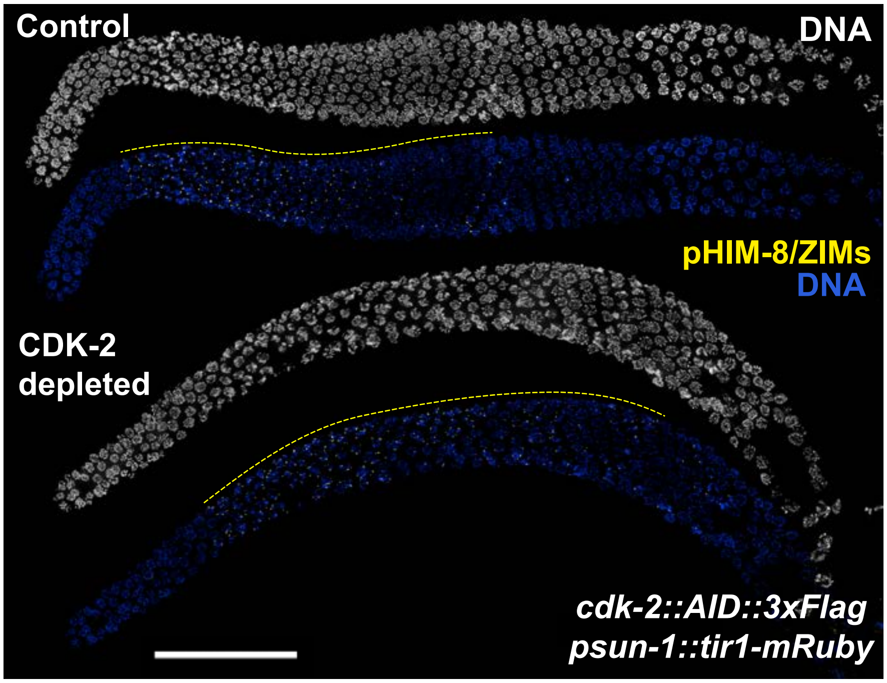

Fig. S3.
(A) Immunofluorescence images of full-length gonads from control (no auxin) and CDK-2-depleted (1 mM auxin treatment for 24 hr) worms. Staining for DNA (white and blue) and pHIM-8/ZIMs (yellow) is shown. pHIM-8/ZIM immunostaining, like DSB-2 immunostaining, is a marker indicative of active CHK-2 protein kinase, which is in turn a marker of meiotic prophase progression. Scale bar, 50 μm. Dotted yellow lines indicate the pHIM-8/ZIM positive regions representing the "CHK-2 active zone", which is extended in the CDK-2 depleted gonad.

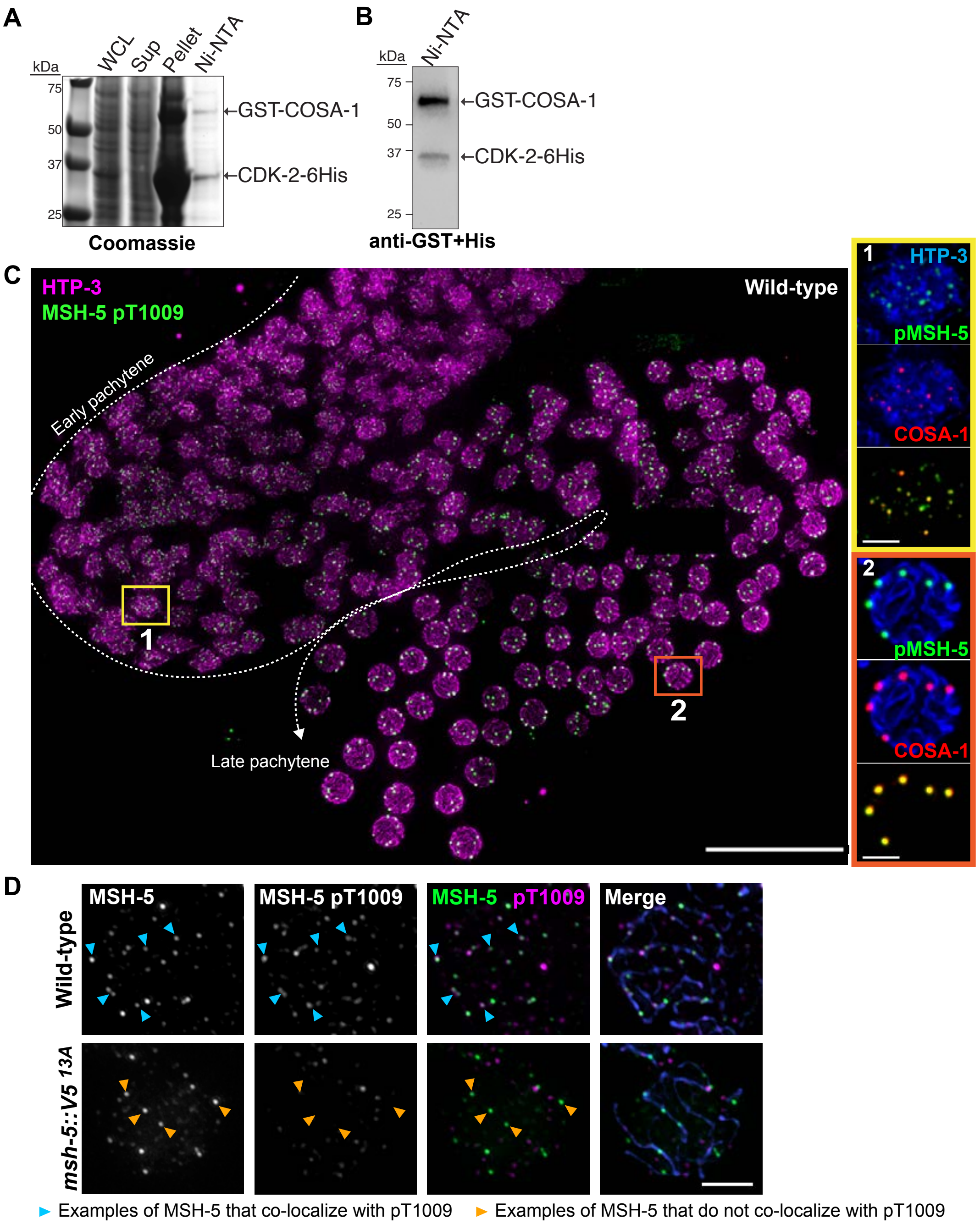

Fig. S4. Evidence for association of recombinant CDK-2 and COSA-1 proteins and characterization of the MSH-5 pT1009 antibody

(A) Coomassie-stained SDS-PAGE gel showing co-enrichment of bands corresponding in size to CDK-2-6His and GST-COSA-1 proteins (coexpressed in insect cells) using Ni-NTA beads. WCL: whole cell lysates; Sup: supernatant. (B) Immunoblot using both GST and His antibodies for detection, showing that GST-COSA-1 and CDK-2-6His are both present in proteins eluted from Ni-NTA beads. (C) Composite immunofluorescence image of a spread wild-type gonad showing HTP-3 (magenta) and MSH-5 pT1009 (green). The direction of meiotic progression is indicated by the white dotted arrow. Scale bar, 15 μm. Insets are representative (1) early and (2) late pachytene nuclei, showing MSH-5 pT1009 (green), COSA-1 (red) and HTP-3 (blue) staining.

Scale bars, 2 μm (D) Immunofluorescence images of late pachytene nuclei from wild-type and *msh-5::V5*^{13A} mutant showing MSH-5 (green), MSH-5 pT1009 (magenta), and HIM-3 (blue). Scale bar, 5 μm. Examples of MSH-5 foci that colocalize with the MSH-5 pT1009 signal are indicated by cyan arrowheads, and MSH-5 foci that do not colocalize with pT1009 antibody signals are indicated by orange arrowheads.

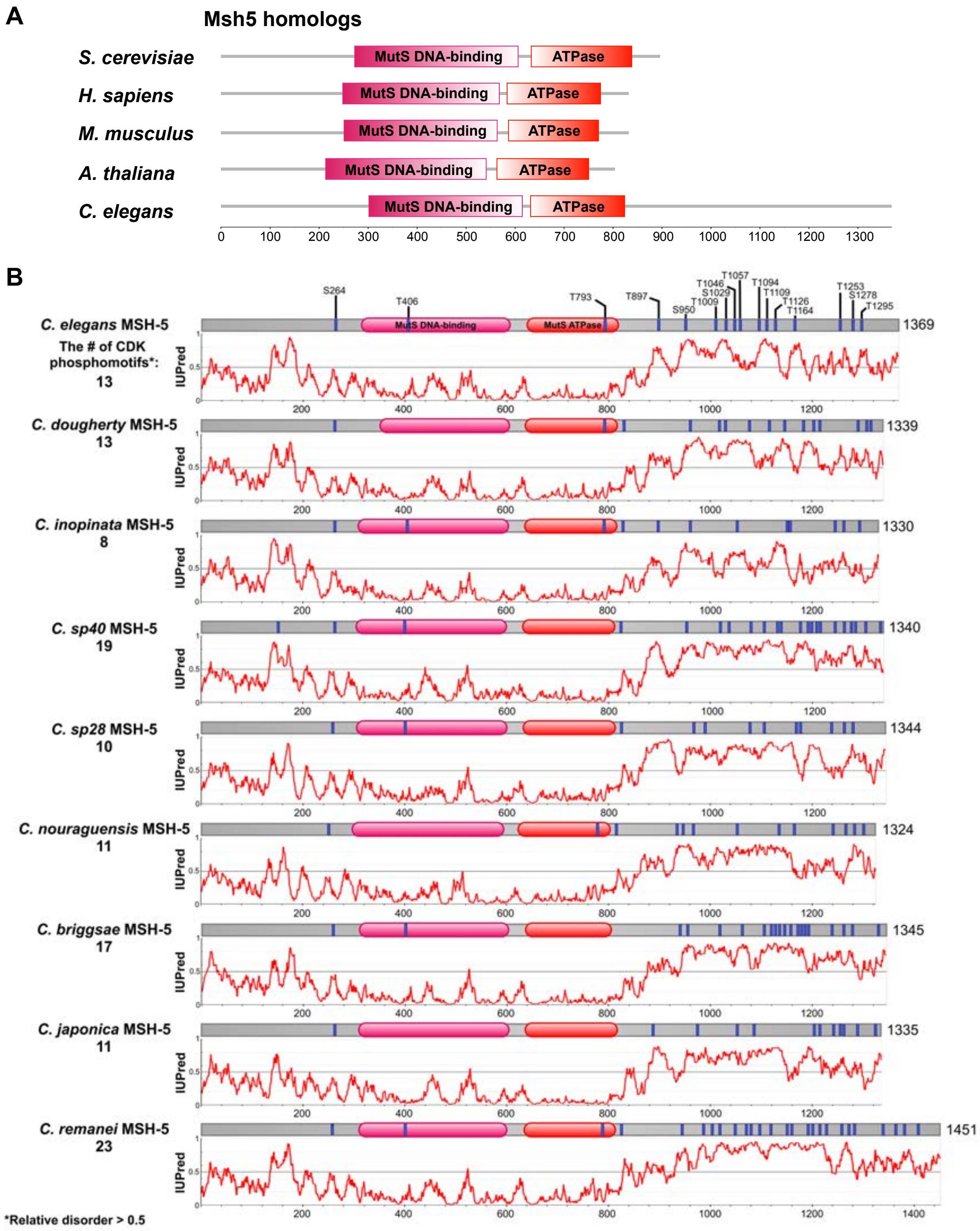

Fig. S5. The domain structure of Msh5 orthologs and the distribution of CDK consensus sites in related *Caenorhabditis* species

(A) Schematic showing the domain structure of Msh5 orthologs from indicated eukaryotic species. (B) Schematic showing predicted CDK phosphorylation sites within MSH-5 orthologs from related *Caenorhabditis* species. The IUPred disorder plots are shown below. The number of CDK consensus motifs within the disordered C-terminal tail is indicated on the left.

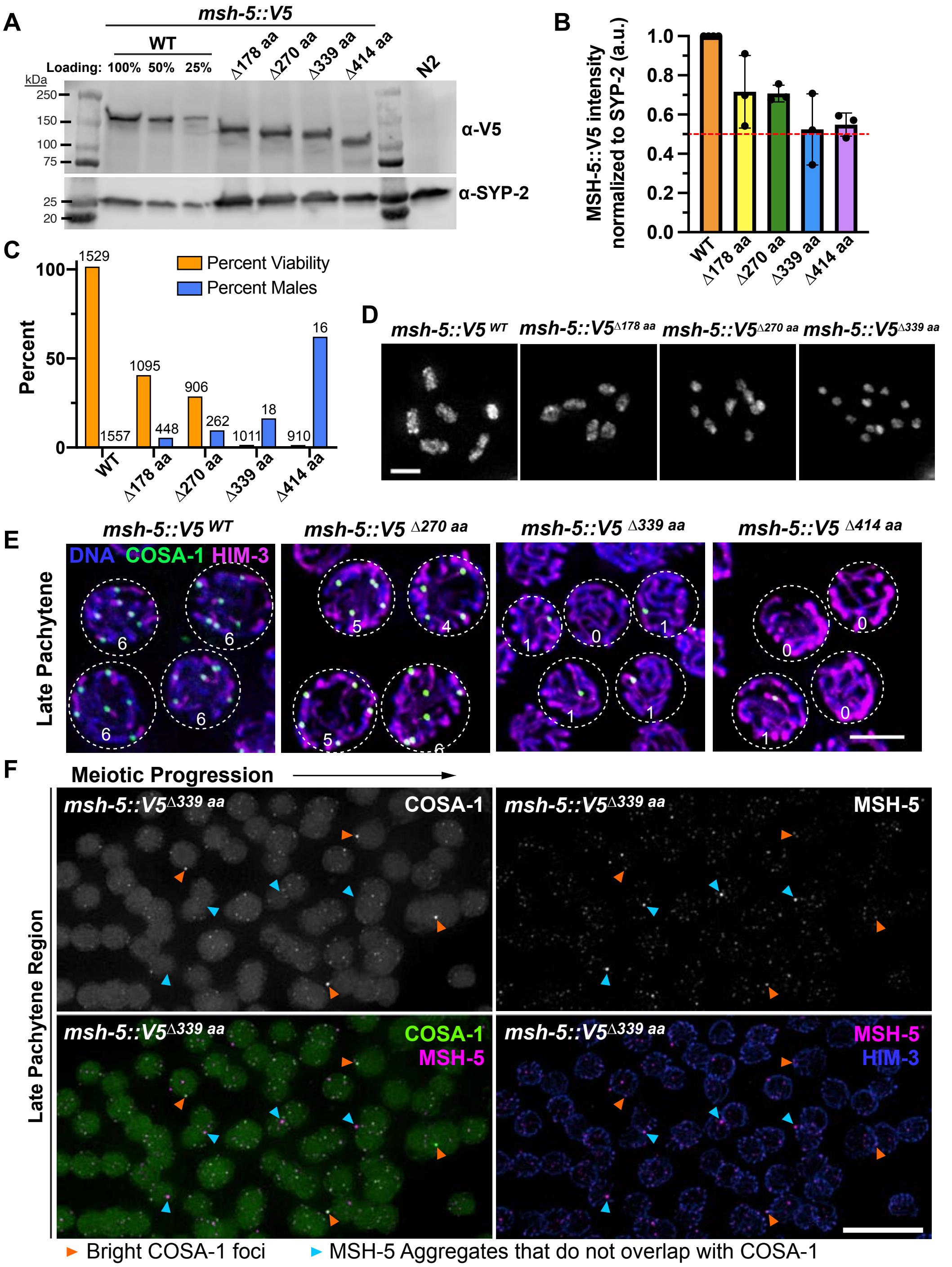

Fig. S6. Analysis of *msh-5* C-terminal truncation mutants

(A) Immunoblot showing the expression of the MSH-5 C-terminal truncation proteins in whole worm lysates. 100%, 50% and 25% of *msh-5::V5*^{WT} lysates were loaded for comparison, and SYP-2 was used as a loading control. (B) Quantification of the band intensity of MSH-5::V5 normalized to SYP-2. The average was measured from 3 independent western blots. Error bars show SD. (C) Graph showing the percent viable self-progeny and males from indicated truncation mutants. Numbers of nuclei scored are shown on the top. (D) Oocyte nuclei at diakinesis from indicated genotypes were stained with DAPI. Scale bar, 3 μm. (E) Immunofluorescence images

of late pachytene nuclei from the indicated genotypes showing DNA (blue), COSA-1 (green), and HIM-3 (magenta) staining. The boundary of individual nuclei is indicated by white dotted lines, and the number of COSA-1 foci in each nucleus is also shown. Scale bar, 3 μm. (F) Immunofluorescence of late pachytene region of a spread gonad from *msh-5::V5*$^{\Delta 339aa}$ showing the staining for COSA-1, MSH-5, and HIM-3. Scale bar, 10 μm.

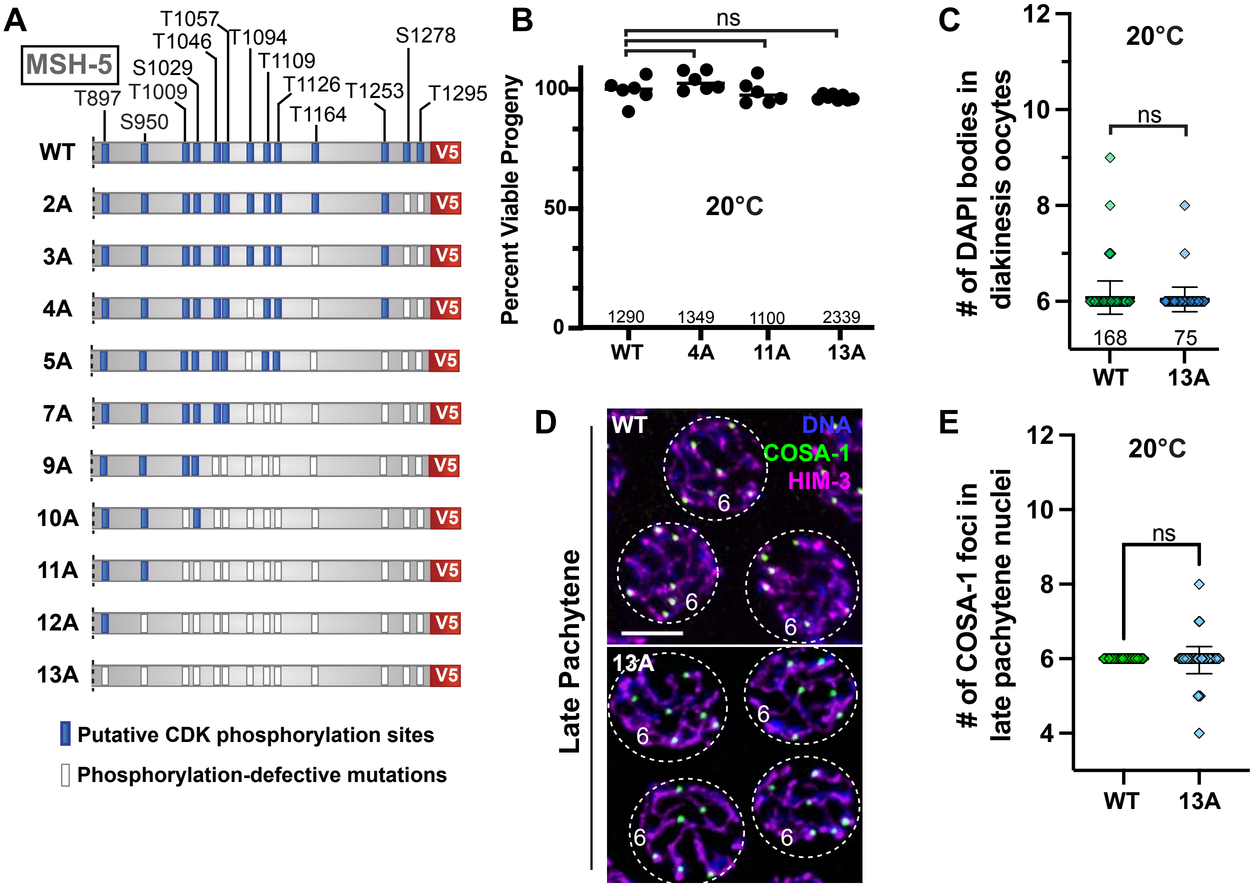

Fig. S7. Mutation of all CDK consensus sites within MSH-5 C-terminal tail does not cause meiotic defects in an otherwise wild-type background

(A) Diagram of phosphorylation-defective *msh-5* alleles generated for this study. (B) Graph showing the viability of self-progeny from the indicated genotypes at 20°C. Numbers of eggs scored are indicated in the bottom. All *msh-5* mutants do not show significant differences from the wild-type. ns, not significant by ordinary one-way ANOVA (*msh-5::V5*^{WT} *vs. msh-5::V5*^{4A}, p = 0.2526; *msh-5::V5*^{WT} *vs. msh-5::V5*^{11A}, p = 0.9935, *msh-5::V5*^{WT} *vs. msh-5::V5*^{13A}, p = 0.4988). (C) Graph showing quantification of DAPI bodies in diakinesis oocytes from *msh-5::V5*^{WT} and *msh-5::V5*^{13A} animals grown at 20°C. Mean ± SD is shown. Numbers of nuclei scored are indicated in the bottom. ns, not significant (p = 0.3240) by Mann-Whitney test. (D) Immunofluorescence images of late pachytene nuclei from *msh-5::V5*^{WT} and *msh-5::V5*^{13A} animals showing DNA (blue), COSA-1 (green), and HIM-3 (magenta) staining. The boundaries of individual nuclei are indicated by white dotted lines, and the number of COSA-1 foci per nucleus is indicated. Scale bar, 5 μm. (E) Graph showing the number of COSA-1 foci in late pachytene nuclei from *msh-5::V5*^{WT} and *msh-5::V5*^{13A} animals. Mean ± SD is shown. Numbers of nuclei scored are indicated in the bottom. ns, not significant (p = 0.1888) by Mann-Whitney test.

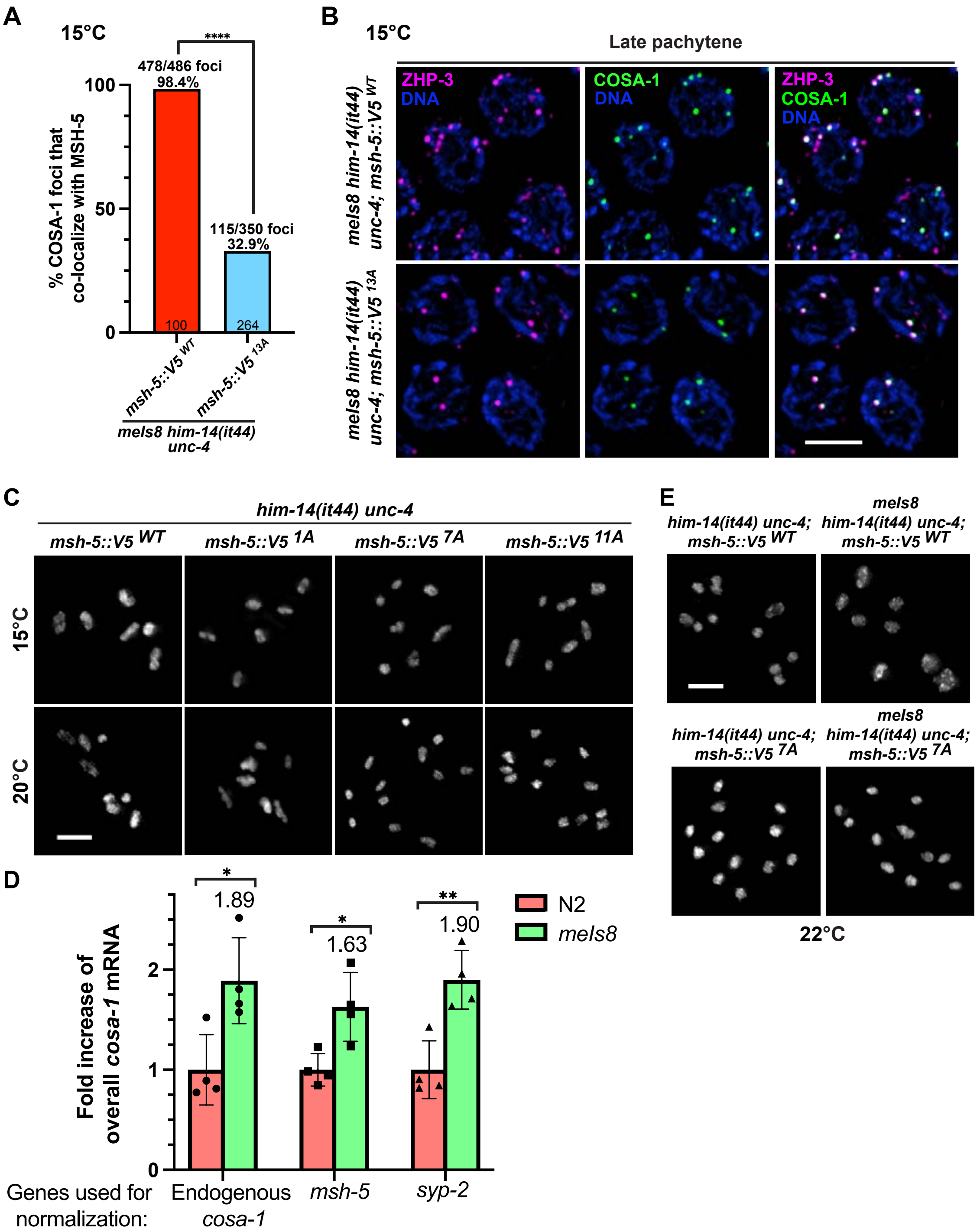

Fig. S8. Localization of other pro-crossover factors in *him-14(it44)* mutants harboring phospho-null mutations of *msh-5*

(A) Graph showing the percentage of COSA-1 foci co-localizing with MSH-5 from germline spreads of indicated genotypes grown at 15°C. Numbers of nuclei scored are shown below, and total foci counted are shown above. ****, p<0.0001 by two-tailed, unpaired t-test. (B) Immunofluorescence images of late pachytene nuclei from indicated genotypes grown at 15°C showing staining for DAPI (blue), ZHP-3 (magenta), and GFP::COSA-1 (green). Scale bar, 3

μm. (C) Oocyte nuclei at diakinesis from indicated genotypes grown at 15°C and 20°C were stained with DAPI. Scale bar, 3 μm. (D) Graph showing the fold increase of overall *cosa-1* mRNA levels in N2 (red) and *meIs8* (green) animals, determined by Taqman qPCR. Three genes (the endogenous *cosa-1, msh-5 and syp-2)* were used for normalization. Error bars are SD from two technical replicates. **, p=0.0047; *, p=0.0164-0.0185 by unpaired t test. (E) DAPI-stained bodies in diakinesis oocytes from indicated genotypes at 22°C. Scale bar, 3 μm.

Supplementary Tables
Table S1 Alleles generated in this study

Allele	Genotype	Information about mutagenesis
kim31	*cdk-2(kim31[cdk-2::AID::3xFLAG]) I*	Generated using CRISPR in *meIs8[pie-1p::GFP::cosa-1 + unc-119(+)] II; ieSi38 [sun-1p::TIR1::mRuby::sun-1 3'UTR + Cbr-unc-119(+)] IV*
kim32	*msh-5(kim32[msh-5::V5]) IV*	Generated using CRISPR in N2
kim34	*msh-5(kim34[msh-5(T1009A)::V5]) IV*	Generated using CRISPR in *msh-5(kim32[msh-5::V5]) IV*
kim35	*msh-5(kim35[msh-5*(S1278A, T1295A)::V5]) IV 2A	Generated using CRISPR in *cdk-2(kim31[cdk-2::AID::3xFLAG]) I; meIs8[pie-1p::GFP::cosa-1, unc-119(+)] II; ieSi38 [sun-1p::TIR1::mRuby::sun-1 3'UTR + Cbr-unc-119(+)] msh-5(kim32[msh-5::V5]) IV*
kim36	*msh-5(kim36[msh-5(*T1164A, S1278A, T1295A)::V5]) IV 3A	Generated using CRISPR in *cdk-2(kim31[cdk-2::AID::3xFLAG]) I; meIs8[pie-1p::GFP::cosa-1, unc-119(+)] II; ieSi38 [sun-1p::TIR1::mRuby::sun-1 3'UTR + Cbr-unc-119(+)] msh-5(kim35[msh-5*(S1278A, T1295A)::V5*]) IV*
kim37	*msh-5(kim37[msh-5*(T1094A, T1164A, S1278A, T1295A)::V5]) IV 4A	Generated using CRISPR in *cdk-2(kim31[cdk-2::AID::3xFLAG]) I; meIs8[pie-1p::GFP::cosa-1, unc-119(+)] II; ieSi38 [sun-1p::TIR1::mRuby::sun-1 3'UTR + Cbr-unc-119(+)] msh-5(kim36[msh-5*(T1164A, S1278A, T1295A)::V5*]) IV*
kim38	*msh-5(kim38[msh-5*(T1094A, T1164A, T1253A, S1278A, T1295A)::V5]) IV 5A	Generated using CRISPR in *cdk-2(kim31[cdk-2::AID::3xFLAG]) I; meIs8[pie-1p::GFP::cosa-1, unc-119(+)] II; ieSi38 [sun-1p::TIR1::mRuby::sun-1 3'UTR + Cbr-unc-119(+)] msh-5(kim37[msh-5*(T1094A, T1164A, S1278A, T1295A)::V5]) *IV*
kim39	*msh-5(kim39[msh-5*(T1094A, T1109A, T1164A, T1253A, S1278A, T1295A)::V5]) IV 6A	Generated using CRISPR in *cdk-2(kim31[cdk-2::AID::3xFLAG]) I; ieSi38 [sun-1p::TIR1::mRuby::sun-1 3'UTR + Cbr-unc-119(+)] msh-5(kim38[msh-5*(T1094A, T1164A, T1253A, S1278A, T1295A)::V5]) *IV*
kim41	*msh-5(kim41[msh-5*(T1094A, T1109A, T1126A, T1164A, T1253A, S1278A, T1295A)::V5]) IV 7A	Generated using CRISPR in *cdk-2(kim31[cdk-2::AID::3xFLAG]) I; ieSi38 [sun-1p::TIR1::mRuby::sun-1 3'UTR + Cbr-unc-119(+)] msh-5(kim38[msh-5*(T1094A, T1164A, T1253A, S1278A, T1295A)::V5]) IV
kim42	*msh-5(kim42[msh-5*(T1046A, T1057A, T1094A, T1164A, T1253A, S1278A, T1295A)::V5]) IV 7A2	Generated using CRISPR in *cdk-2(kim31[cdk-2::AID::3xFLAG]) I; ieSi38 [sun-1p::TIR1::mRuby::sun-1 3'UTR + Cbr-unc-119(+)] msh-5(kim38[msh-5*(T1094A, T1164A, T1253A, S1278A, T1295A)::V5]) *IV*
kim44	*msh-5(kim44[msh-5*(T1046A, T1057A, T1094A, T1109A, T1126A, T1164A, T1253A, S1278A, T1295A)::V5]) IV 9A	Generated using CRISPR in *cdk-2(kim31[cdk-2::AID::3xFLAG]) I; ieSi38 [sun-1p::TIR1::mRuby::sun-1 3'UTR + Cbr-unc-119(+)] msh-5(kim42[msh-5*(T1046A, T1057A, T1094A, T1164A, T1253A, S1278A, T1295A)::V5]) *IV*
kim45	*msh-5(kim45[msh-5*(T1009A, T1046A, T1057A, T1094A, T1109A, T1126A, T1164A, T1253A, S1278A, T1295A)::V5]) IV 10A	Generated using CRISPR in *cdk-2(kim31[cdk-2::AID::3xFLAG]) I; ieSi38 [sun-1p::TIR1::mRuby::sun-1 3'UTR + Cbr-unc-119(+)] msh-5(kim44[msh-5*(T1046A, T1057A, T1094A, T1109A, T1126A, T1164A, T1253A, S1278A, T1295A)::V5]) *IV*
kim47	*msh-5(kim47[msh-5*(T1009A, S1029A, T1046A, T1057A, T1094A, T1109A, T1126A, T1164A, T1253A, S1278A, T1295A)::V5]) *IV* 11A	Generated using CRISPR in *cdk-2(kim31[cdk-2::AID::3xFLAG]) I; ieSi38 [sun-1p::TIR1::mRuby::sun-1 3'UTR + Cbr-unc-119(+)] msh-5(kim45[msh-5*(T1009A, T1046A, T1057A, T1094A, T1109A, T1126A, T1164A, T1253A, S1278A, T1295A)::V5]) *IV*

kim59	*msh-5(kim59[msh-5*(S950A, T1009A, S1029A, T1046A, T1057A, T1094A, T1109A, T1126A, T1164A, T1253A, S1278A, T1295A)::V5]) *IV* 12A	Generated using CRISPR in *msh-5(kim47[msh-5*(T1009A, S1029A, T1046A, T1057A, T1094A, T1109A, T1126A, T1164A, T1253A, S1278A, T1295A)::V5]) IV
kim60	*msh-5(kim60[msh-5*(T897A, S950A, T1009A, S1029A, T1046A, T1057A, T1094A, T1109A, T1126A, T1164A, T1253A, S1278A, T1295A)::V5]) *IV* 13A	Generated using CRISPR in *msh-5(kim59[msh-5*(S950, T1009A, S1029A, T1046A, T1057A, T1094A, T1109A, T1126A, T1164A, T1253A, S1278A, T1295A)::V5]) IV
kim48	*msh-5(kim48[msh-5*(aa 1-1191)::V5]) *IV*	Generated using CRISPR in *cdk-2(kim31[cdk-2::AID::3xFLAG]) I; meIs8[pie-1p::GFP::cosa-1, unc-119(+)] II; ieSi38 [sun-1p::TIR1::mRuby::sun-1 3'UTR + Cbr-unc-119(+)] him-6(jf93[him-6::HA]) msh-5(kim32[msh-5::V5]) IV*
kim49	*msh-5(kim49[msh-5*(aa 1-1099)::V5]) *IV*	Generated using CRISPR in *cdk-2(kim31[cdk-2::AID::3xFLAG]) I; meIs8[pie-1p::GFP::cosa-1, unc-119(+)] II; ieSi38 [sun-1p::TIR1::mRuby::sun-1 3'UTR + Cbr-unc-119(+)] him-6(jf93[him-6::HA]) msh-5(kim32[msh-5::V5]) IV*
kim50	*msh-5(kim50[msh-5*(aa 1-1030)::V5]) *IV*	Generated using CRISPR in *cdk-2(kim31[cdk-2::AID::3xFLAG]) I; meIs8[pie-1p::GFP::cosa-1, unc-119(+)] II; ieSi38 [sun-1p::TIR1::mRuby::sun-1 3'UTR + Cbr-unc-119(+)] him-6(jf93[him-6::HA]) msh-5(kim32[msh-5::V5]) IV*
kim51	*msh-5(kim51[msh-5*(aa 1-955)::V5]) *IV*	Generated using CRISPR in *cdk-2(kim31[cdk-2::AID::3xFLAG]) I; meIs8[pie-1p::GFP::cosa-1, unc-119(+)] II; ieSi38 [sun-1p::TIR1::mRuby::sun-1 3'UTR + Cbr-unc-119(+)] him-6(jf93[him-6::HA]) msh-5(kim32[msh-5::V5]) IV*
kim63	*zhp-3(kim63[zhp-3::V5]) I*	Generated using CRISPR in *cdk-2(kim31[cdk-2::AID::3xFLAG]) I; meIs8[pie-1p::GFP::cosa-1, unc-119(+)] II; ieSi38 [sun-1p::TIR1::mRuby::sun-1 3'UTR + Cbr-unc-119(+)]) IV*
kim64	*zhp-3(kim63[zhp-3::ollas]) I*	Generated using CRISPR in *cdk-2(kim31[cdk-2::AID::3xFLAG]) I; meIs8[pie-1p::GFP::cosa-1, unc-119(+)] II; ieSi38 [sun-1p::TIR1::mRuby::sun-1 3'UTR + Cbr-unc-119(+)]) IV*

Table S2

crRNAs, repair templates and genotyping primers for mutant alleles generated in this study

Allele	crRNAs and repair templates	Genotyping primers and fragment sizes
kim31 *(cdk-2::AID::3x FLAG)*	5'- TCCTGATGATTGTTCAGCAG-3'; 5'-GCGCAGAACAGGgtaaagttactggaatatttggatatttttttaatgaaaaaaaattatttcagACGCCACCGCTtCTcAAtAAcCAcCAaGAGAAGTCAATCTTCGGAGGCTCAGGAatgcctaaagatccagccaaacctccggccaaggcacaagttgtgggatggccaccggtgagatcataccggaagaacgtgatggtttcctgccaaaaatcaagcggtggcccggaggcggcggcgttcgtgaagGGATCGGACTATAAAGATCACGACGGAGATTACAAGGACCATGATATCGACTACAAGGACGACGACGACAAGGGATAAtatatcatgtcctccataacctaaacatcgtgtactatgtcctctcttttaccgagtctttcctc-3'	F 5'- GAATAATTTTTGCAGAGCTGGCC-3'; R 5'-GAGGAAAGACTCGGTAAAAGAGAGG-3'; WT, 440 bp; Mutant, 660 bp
kim32 *(msh-5::V5)*	5'- CGAACGATCTATCGTCTCAT -3'; 5'-TGAAACAGAAGGATCTCTCCATATCGATACGAGCGCtGATGAGACGATAGATCGTTCGAAAAGAAGTGGATCGGGAAAGCCAATTCCAAACCCACTTCTTGGACTCGACTCCACCTAAttttatataattagaatttcgtatttcttgtaatg -3'	F 5'- AAAAACGTCCCACAGAAGC -3'; R 5'-CGAAATACATTGAACAAGATTGA -3'; WT, 433 bp; Mutant, 481 bp
kim34 and kim45 (*T1009A*)	5'- TCACCCATTTGGATTGGAGT -3'; 5'-GATCTAGGCCTTTTGACACCACCAGCTTCtCCCATcTGaATaGGtGccGGAATATGTATTGCAGTTCGAGTCGAGAATGAGTG -3'	F 5'- TTCCGGCACCTATTCAGATGGGA -3'; R 5'-TTGGTCCCAGCACTGATCTCCAA -3'; WT, No band; Mutant, 378 bp
kim35 (*S1278A, T1295A*)	5'-ATTCTGGGACAACTTGCTTT-3'; 5'-AATAAAGGACAAGCTTCAAATAGTTCGATTgCtCCGAGCAGTCTGATcCTcGGtCAgCTcGCcTTcGGtGACGTCGATCAAgCtCCTCGTCCTCGTGGGGACAATCCAATTGAA-3'	F 5'- CCTCGGTCAGCTCGCCTT -3'; R 5'-ATTGGCTTTCCCGATCCACT -3'; WT, No band; Mutant, 276 bp
kim36 (*T1164A*)	5'-GATGATCTTGGAGTGACATT-3'; 5'-TCGATTCTGAAATCACAAGATACATATGATCCAAAcGTcgCaCCtcGtagcagcTCTCGTCGTGAACTCAGACCGGATGTGTCT-3'	F 5'- AAACGTCGCACCTCGTAGCAGC -3'; R 5'- GCTTTTCCGAGCTTCTGGGT -3'; WT, No band; Mutant, 302 bp
kim37 (*T1094A*)	5'- AGACGCCGATCAGTGATAGA -3'; 5'-GAACGATATTTGCAAAGTGATCCATTCAAGgCtCCaATtAGcGAcAGgAGgttccatttttcaattcaattttctattttg -3'	F 5'- GCTCCAATTAGCGACAGGAGG-3'; R 5'-CCGCCAAATACCTAATGAGACC-3'; WT, No band; Mutant, 306 bp
kim38 (T1253A)	5'- CTTTTCCGAGCTTCTGGGTT -3'; 5'-aggttctttaaaattaataacCTTTGCTTTTCCGAagacCgcGGcTCtGGtGccTTaAAgATAAAATTATCAGAGTTGACAATGGGCATCGAGGT -3'	F 5'- CTTTAAGGCACCAGAGCCGC -3'; R 5'-CAGAAAACAGTCTCAATGCACCA -3'; WT, No band; Mutant, 364 bp
kim39 (T1109A)	5'- ATCGATTTTTTGGAGTACTG -3'; 5'-GCACTCTGAATCAATGATTGATTCATTGATCGATTcTTcGGtGcggaaTGtCtCGAAGATTGTTGAGACctgaaatgttgaaattgtg -3'	F 5'- TCGAGACATTCCGCACCGAAGAA -3'; R 5'-CGGCTCTGGTGCCTTAAAGAT -3'; WT, No band; Mutant, 456 bp
kim41 (T1109A, T1126A)	5'- ATCGATTTTTTGGAGTACTG -3'; 5'-CACTTCATTTGAAGATCGAATTGTTTCATGAGGAGcgTCaCgaGCggatTGgATgAgactcTGgTTCATgctcCtgTTcTTcGGtGcggaaTGaCGCGAAGATTGTTGAGACctgaaatgttgaaattg -3'	F 5'- TCTCATCCAATCCGCTCGTGACG -3'; R 5'-GAAGACCGCGGCTCTGGTG -3'; WT, No band; Mutant, 423 bp

kim42 (T1046A, T1057A)	5’- ACTTGTGATTCTTTCACATT -3’; 5’- gcttccaaagcccacCTTATGGAAAGCTGTTTTGGaGctTCaAGgACcTGactcTCcTTgACgTTcGGaGcCTTCTTGAAAACTTCAGTTCTGACTGATTTTGATG -3’	F 5’- GCTCCGAACGTCAAGGAGAGTCA -3’; R 5’- CCTAATGAGACCCAGCGGTTGGA -3’; WT, No band; Mutant, 487 bp
kim44 (T1109A, T1126A)	5’- ATCGATTTTTTGGAGTACTG -3’; 5’- CACTTCATTTGAAGATCGAATTGTTTCATGAGGAGcgTCaCgaGCggatTGgATgAgactcTGgTTCATgctcCtgTTcTTcGGtGcggaaTGaCGCGAAGATTGTTGAGACctgaaatgttgaaattg -3’	F 5’- TCTCATCCAATCCGCTCGTGACG -3’; R 5’- GAAGACCGCGGCTCTGGTG -3’; WT, No band; Mutant, 423 bp
kim46 (A1109T in 11A)	5’- CATgctcCtgTTcTTcGGtG -3’; 5’- gatTGgATgAgactcTGgTTCATgctcCtgTTcTTtGGtGtactgTGtCtaGAAGATTGTTGAGACctgaaatgttgaaattgtga -3’	F 5’- TCCAACCGCTGGGTCTCATTAGG -3’; R 5’- CTTTGGTGTACTGTGTCTAGAAG -3’; WT, No band; Mutant, 508 bp
kim47 (S1029A)	5’- TCAACGTCTACTTCATCACC -3’; 5’- AgATGGGaGAAGCTGGTGGTGTCAAAAGGCCTAGAagcACcagcACcagtgCtCCtGGTCCTTCAGCATCAAAATCAGTCAGAACTGAAGTTT -3’	F 5’- TAATCGAAAGGGCCGAACTTT -3’; R 5’- GAGCACTGGTGCTGGTGCT -3’; WT, No band; Mutant, 553 bp
kim59 (S950A)	5’- TCCTCTTCTTGGAATGGATC -3’; 5’- GTGACAGGTAGTAGTATGGAATCATCAATGgCTCCaGAcCCgTTtCAgGAgGAaGAcGAAGGTACTGAAGGAGAGGAAGATCAAATA -3’	F 5’- GAAGATGGTTTCATGGCCG-3’; R 5’- TCCTCCTGAAACGGGTCTG -3’; WT, No band; Mutant, 345 bp
kim60 (T897A)	5’- GAGATCAGAAGCTTCTACTC -3’; 5’- GAGCGAGGAAATTGAAAAGGAGAGATCAGAgGCaagcgCcCCaGCtTCGAAAAGTCGCAGTACCATCACAGCCAGg -3’	F 5’- TTGCCGGTTTGCCGATTTGC -3’; R 5’- AAGCTGGGGCGCTTGCCT -3’; WT, No band; Mutant, 846 bp
kim48 *msh-5* (aa 1-1191) ::V5	5’- TGGCGAAGTATTCTCTGAGT -3’; 5’- CTCAGAATAGTCAATTTGGCGAAGTATTCTCTGAGGGATCGGGAAAGCCAATTCCAAACCCACTTCTTGGACTCGACTCCACCTAATTaGGCACACAGTTCTCCATATTCAATAGTCAACAGTCA -3’	F 5’- TCGTGAACTCAGACCGGATG -3’; R 5’- TGACAATGGGCATCGAGGTT -3’; WT, 224 bp; Mutant, 275 bp
kim49 *msh-5* (aa 1-1099) ::V5	5’- AGACGCCGATCAGTGATAGA -3’; 5’- AAAGTGATCCATTCAAGACGCCGATCAGTGATAGAGGATCGGGAAAGCCAATTCCAAACCCACTTCTTGGACTCGACTCCACCTAAAGattccatttttcaattcaattttctattttggagat -3’	F 5’- AGTGATCCATTCAAGACGCC -3’; R 5’- AAAAAGACTCACTTTCTGTAAGCC -3’; WT, 233 bp; Mutant, 284 bp
kim50 *msh-5* (aa 1-1030) ::V5	5’- TCAACGTCTACTTCATCACC -3’; 5’- GCTGGTGGTGTCAAAAGGCCTAGATCAACGTCTACCcTCcTCtCCtGGATCGGGAAAGCCAATTCCAAACCCACTTCTTGGACTCGACTCCACCTAAGGTCCTTCAGCATCAAAATCAGTCAGAACTGAAGT -3’	F 5’- GGTGAAGCTGGTGGTGTCAA -3’; R 5’- GGCTTCCAAAGCCCACCTTAT -3’; WT, 168 bp; Mutant, 219 bp
kim51 *msh-5* (aa 1-955) ::V5	5’- TCCTGATCCATTCCAAGAAG -3’; 5’- GTATGGAATCATCAATGTCTCCTGATCCATTCCAAGGATCGGGAAAGCCAATTCCAAACCCACTTCTTGGACTCGACTCCACCTAAGAAGAaGATGAAGGTACTGAAGGAGAGGAAGATCAAATAAG -3’	F 5’- CACAGCCAGGTTATTACTGTGA -3’; R 5’- ACAGACGGAAGTGTTGGTCG -3’; WT, 268 bp; Mutant, 319 bp
kim63 (*zhp-3::V5*)	5’- ATTAAtgttttaatctcgtt -3’; 5’- GGAAACCGATCAATGGTCGGAGCTTCATTGGACCCGCCGATGGATCGGGAAAGCCAATTCCAAACCCACTTCTTGGACTCGACTCCACCTAAtgttttaatctcgtttttttctgaattcgttctttatttg -3’	F 5’- CCTTTCCGCTCCCTCTATATTC -3’; R 5’- ATCAGATGTGAACTAGGTAGAGA -3’; WT, 263 bp; Mutant, 311 bp
kim64 (*zhp-3::ollas*)	5’- aacgagattaaaacaTTAAT -3’; 5’- AAACCGATCAATGGTCGGAGCTTCATTGGACCCGCtGATGGATCGagcggattcgctaacgaac	F 5’- CCTTTCCGCTCCCTCTATATTC -3’; R 5’- ATCAGATGTGAACTAGGTAGAGA -3’; WT, 263 bp; Mutant, 311 bp

	tcggaccacgcctcatgggaaagTAAtgttttaatctcgtttttt ctgaattcgttc -3’	

Table S3

Strains used in this study

Strains	Source	Strain #
N2	Caenorhabditis Genetics Center	CB
meIs8[pie-1p::GFP::cosa-1, unc-119(+)] II	Yokoo et al., 2012	AV630
cdk-2(kim31[cdk-2::AID::3xFLAG]) I; meIs8[pie-1p::GFP::cosa-1, unc-119(+)] II; ieSi38 [sun-1p::TIR1::mRuby::sun-1 3'UTR + Cbr-unc-119(+)] IV	This study	YKIM110
cdk-2(kim31[cdk-2::AID::3xFLAG]) I; cosa-1(tm3298)/qC1[qIs26] III; ieSi38 [sun-1p::TIR1::mRuby::sun-1 3'UTR + Cbr-unc-119(+)] IV	This study	YKM323
him-6(jf93[him-6::HA]) IV	Jagut et al., 2016	UV116
cdk-2(kim31[cdk-2::AID::3xFLAG]) I; meIs8[pie-1p::GFP::cosa-1, unc-119(+)] II; ieSi38 [sun-1p::TIR1::mRuby::sun-1 3'UTR + Cbr-unc-119(+)] him-6(jf93[him-6::HA]) msh-5(kim32[msh-5::V5]) IV	This study	YKM762
cdk-2(kim31[cdk-2::AID::3xFLAG]) I; meIs8[pie-1p::GFP::cosa-1, unc-119(+)] II; ieSi38 [sun-1p::TIR1::mRuby::sun-1 3'UTR + Cbr-unc-119(+)] msh-5(kim32[msh-5::V5]) IV	This study	YKM306
msh-5(kim32[msh-5::V5]) IV	This study	YKM154
him-14(it44) unc-4(e120)/mnC1 II	Girard et al., 2021	KK323
meIs8[pie-1p::GFP::cosa-1, unc-119(+)] him-14(it44) unc-4(e120)/mnC1 II; oxTi602 IV	This study	AV1176
him-14(it44) unc-4(e120)/mnC1 II; msh-5(kim32[msh-5::V5]) IV	This study	AV1172
meIs8[pie-1p::GFP::cosa-1, unc-119(+)] him-14(it44) unc-4(e120)/mnC1 II; msh-5(kim32[msh-5::V5]) IV	This study	AV1171
msh-5(kim34[msh-5(T1009A)::V5]) IV	This study	YKM233
him-14(it44) unc-4(e120)/mnC1 II; msh-5(kim34[msh-5(T1009A)::V5]) IV	This study	AV1166
meIs8[pie-1p::GFP::cosa-1, unc-119(+)] him-14(it44) unc-4(e120)/mnC1 II; msh-5(kim34[msh-5(T1009A)::V5]) IV	This study	AV1165
cdk-2(kim31[cdk-2::AID::3xFLAG]) I; meIs8[pie-1p::GFP::cosa-1, unc-119(+)] II; ieSi38 [sun-1p::TIR1::mRuby::sun-1 3'UTR + Cbr-unc-119(+)] msh-5(kim35[msh-5(S1278A, T1295A)::V5*]) IV*	This study	YKM398
cdk-2(kim31[cdk-2::AID::3xFLAG]) I; meIs8[pie-1p::GFP::cosa-1, unc-119(+)] II; ieSi38 [sun-1p::TIR1::mRuby::sun-1 3'UTR + Cbr-unc-119(+)] msh-5(kim36[msh-5(T1164A, S1278A, T1295A)::V5*]) IV*	This study	YKM459
cdk-2(kim31[cdk-2::AID::3xFLAG]) I; meIs8[pie-1p::GFP::cosa-1, unc-119(+)] II; ieSi38 [sun-1p::TIR1::mRuby::sun-1 3'UTR + Cbr-unc-119(+)] msh-5(kim37[msh-5(T1094A, T1164A, S1278A, T1295A)::V5]) *IV*	This study	YKM477
cdk-2(kim31[cdk-2::AID::3xFLAG]) I; meIs8[pie-1p::GFP::cosa-1, unc-119(+)] II; ieSi38 [sun-1p::TIR1::mRuby::sun-1 3'UTR + Cbr-unc-119(+)] msh-5(kim38[msh-5(T1094A, T1164A, T1253A, S1278A, T1295A)::V5]) *IV*	This study	YKM460
cdk-2(kim31[cdk-2::AID::3xFLAG]) I; ieSi38 [sun-1p::TIR1::mRuby::sun-1 3'UTR + Cbr-unc-119(+)] msh-5(kim38[msh-5(T1094A, T1164A, T1253A, S1278A, T1295A)::V5]) *IV*	This study	YKM478

cdk-2(kim31[cdk-2::AID::3xFLAG]) I; ieSi38 [sun-1p::TIR1::mRuby::sun-1 3'UTR + Cbr-unc-119(+)] msh-5(kim39[msh-5(T1094A, T1109A, T1164A, T1253A, S1278A, T1295A)::V5]) *IV*	This study	YKM535
cdk-2(kim31[cdk-2::AID::3xFLAG]) I; ieSi38 [sun-1p::TIR1::mRuby::sun-1 3'UTR + Cbr-unc-119(+)] msh-5(kim41[msh-5(T1094A, T1109A, T1126A, T1164A, T1253A, S1278A, T1295A)::V5]) IV	This study	YKM532
him-14(it44) unc-4(e120)/mnC1 II; msh-5(kim41[msh-5(T1094A, T1109A, T1126A, T1164A, T1253A, S1278A, T1295A)::V5]) IV	This study	AV1168
meIs8[pie-1p::GFP::cosa-1, unc-119(+)] him-14(it44) unc-4(e120)/mnC1 II; msh-5(kim41[msh-5(T1094A, T1109A, T1126A, T1164A, T1253A, S1278A, T1295A)::V5]) IV	This study	AV1167
cdk-2(kim31[cdk-2::AID::3xFLAG]) I; ieSi38 [sun-1p::TIR1::mRuby::sun-1 3'UTR + Cbr-unc-119(+)] msh-5(kim42[msh-5(T1046A, T1057A, T1094A, T1164A, T1253A, S1278A, T1295A)::V5]) *IV*	This study	YKM556
cdk-2(kim31[cdk-2::AID::3xFLAG]) I; ieSi38 [sun-1p::TIR1::mRuby::sun-1 3'UTR + Cbr-unc-119(+)] msh-5(kim44[msh-5(T1046A, T1057A, T1094A, T1109A, T1126A, T1164A, T1253A, S1278A, T1295A)::V5]) *IV*	This study	YKM505
cdk-2(kim31[cdk-2::AID::3xFLAG]) I; ieSi38 [sun-1p::TIR1::mRuby::sun-1 3'UTR + Cbr-unc-119(+)] msh-5(kim45[msh-5(T1009A, T1046A, T1057A, T1094A, T1109A, T1126A, T1164A, T1253A, S1278A, T1295A)::V5]) *IV*	This study	YKM506
cdk-2(kim31[cdk-2::AID::3xFLAG]) I; ieSi38 [sun-1p::TIR1::mRuby::sun-1 3'UTR + Cbr-unc-119(+)] msh-5(kim47[msh-5(T1009A, S1029A, T1046A, T1057A, T1094A, T1109A, T1126A, T1164A, T1253A, S1278A, T1295A)::V5]) *IV*	This study	YKM507
msh-5(kim47[msh-5(T1009A, S1029A, T1046A, T1057A, T1094A, T1109A, T1126A, T1164A, T1253A, S1278A, T1295A)::V5]) *IV*	This study	YKM655
him-14(it44) unc-4(e120)/mnC1 II; msh-5(kim47[msh-5(T1009A, S1029A, T1046A, T1057A, T1094A, T1109A, T1126A, T1164A, T1253A, S1278A, T1295A)::V5]) *IV*	This study	AV1174
meIs8[pie-1p::GFP::cosa-1, unc-119(+)] him-14(it44) unc-4(e120)/mnC1 II; msh-5(kim47[msh-5(T1009A, S1029A, T1046A, T1057A, T1094A, T1109A, T1126A, T1164A, T1253A, S1278A, T1295A)::V5]) *IV*	This study	AV1173
msh-5(kim59[msh-5(S950A, T1009A, S1029A, T1046A, T1057A, T1094A, T1109A, T1126A, T1164A, T1253A, S1278A, T1295A)::V5]) *IV*	This study	YKM685
msh-5(kim60[msh-5(T897A, S950A, T1009A, S1029A, T1046A, T1057A, T1094A, T1109A, T1126A, T1164A, T1253A, S1278A, T1295A)::V5]) *IV*	This study	YKM692
rmeIs8[pie-1p::GFP::cosa-1, unc-119(+)] II; msh-5(kim60[msh-5(T897A, S950A, T1009A, S1029A, T1046A, T1057A, T1094A, T1109A, T1126A, T1164A, T1253A, S1278A, T1295A)::V5]) *IV*	This study	YKM719
him-14(it44) unc-4(e120)/mnC1 II; msh-5(kim60[msh-5(T897A, S950A, T1009A, S1029A, T1046A, T1057A, T1094A, T1109A, T1126A, T1164A, T1253A, S1278A, T1295A)::V5]) *IV*	This study	AV1170
meIs8[pie-1p::GFP::cosa-1, unc-119(+)] him-14(it44) unc-4(e120)/mnC1 II; msh-5(kim60[msh-5(T897A, S950A, T1009A, S1029A, T1046A, T1057A, T1094A, T1109A, T1126A, T1164A, T1253A, S1278A, T1295A)::V5]) *IV*	This study	AV1169

cdk-2(kim31[cdk-2::AID::3xFLAG]) I; meIs8[pie-1p::GFP::cosa-1, unc-119(+)] II; ieSi38 [sun-1p::TIR1::mRuby::sun-1 3'UTR + Cbr-unc-119(+)] him-6(jf93[him-6::HA]) msh-5(kim48[msh-5(aa 1-1191)::V5]) *IV*	This study	YKM357
cdk-2(kim31[cdk-2::AID::3xFLAG]) I; meIs8[pie-1p::GFP::cosa-1, unc-119(+)] II; ieSi38 [sun-1p::TIR1::mRuby::sun-1 3'UTR + Cbr-unc-119(+)] him-6(jf93[him-6::HA]) msh-5(kim49[msh-5(aa 1-1099)::V5]) *IV*	This study	YKM454
cdk-2(kim31[cdk-2::AID::3xFLAG]) I; meIs8[pie-1p::GFP::cosa-1, unc-119(+)] II; ieSi38 [sun-1p::TIR1::mRuby::sun-1 3'UTR + Cbr-unc-119(+)] him-6(jf93[him-6::HA]) msh-5(kim50[msh-5(aa 1-1030)::V5]) *IV/nT1 [unc-?(n754) let-?] (IV;V)*	This study	YKM401
cdk-2(kim31[cdk-2::AID::3xFLAG]) I; meIs8[pie-1p::GFP::cosa-1, unc-119(+)] II; ieSi38 [sun-1p::TIR1::mRuby::sun-1 3'UTR + Cbr-unc-119(+)] him-6(jf93[him-6::HA]) msh-5(kim51[msh-5(aa 1-955)::V5]) *IV/nT1 [unc-?(n754) let-?] (IV;V)*	This study	YKM402
cdk-2(kim31[cdk-2::AID::3xFLAG]) zhp-3(kim63[zhp-3::V5]) I; meIs8[pie-1p::GFP::cosa-1, unc-119(+)] II; ieSi38 [sun-1p::TIR1::mRuby::sun-1 3'UTR + Cbr-unc-119(+)] IV	This study	YKM165
cdk-2(kim31[cdk-2::AID::3xFLAG]) zhp-3(kim63[zhp-3::ollas]) I; meIs8[pie-1p::GFP::cosa-1, unc-119(+)] II; ieSi38 [sun-1p::TIR1::mRuby::sun-1 3'UTR + Cbr-unc-119(+)] msh-5(kim32[msh-5::V5]) IV	This study	YKM760
cdk-2(kim31[cdk-2::AID::3xFLAG]) zhp-3(kim63[zhp-3::ollas]) I; meIs8[pie-1p::GFP::cosa-1, unc-119(+)] II; ieSi38 [sun-1p::TIR1::mRuby::sun-1 3'UTR + Cbr-unc-119(+)] him-6(jf93[him-6::HA]) msh-5(kim50[msh-5(aa 1-1030)::V5]) *IV/ nT1 [unc-?(n754) let-?] (IV;V)*	This study	YKM761

Table S4

Plasmids used in this study

Plasmid number	Description	Bacterial selection	Figure
pYK301	pFastBac1-CDK-2-6His	Ampicillin	S4A-B
pYK317	pFastBac1-GST-COSA-1	Ampicillin	S4A-B

Table S5

Primers used for quantitative RT-PCR

Name	Sequence	Description
oYK1697	TCAAGTTCTCGGTCACACC	*cosa-1* (F) to detect both the endogenous *cosa-1* and *gfp::cosa-1* transgene
oYK1698	CATTGTCGCTTTTTGGCTCA	*cosa-1* (R) to detect both the endogenous *cosa-1* and the *gfp::cosa-1* transgene
oYK1686	TCGTGAAAACTGAACTGAAGTGT	*cosa-1* (Forward) to detect the endogenous *cosa-1* only
oYK1687	GTTTTTGCGGTGTGACCGAG	*cosa-1* (Reverse) to detect the endogenous *cosa-1* only
oYK1688	CACAAGGGAGCTCAGGATGG	*msh-5* (Forward)
oYK1689	CGGATCCATCGGCATGTCTTT	*msh-5* (Reverse)
oYK1690	CCCAAGATGACCGGAGAGTC	*syp-2* (Forward)
oYK1691	GGTTGTTTAGTTTGGGCCGC	*syp-2* (Reverse)

SI References

1. L. Zhang, J. D. Ward, Z. Cheng, A. F. Dernburg, The auxin-inducible degradation (AID) system enables versatile conditional protein depletion in C. elegans. *Development* **142**, 4374-4384 (2015).
2. M. E. Hurlock *et al.*, Identification of novel synaptonemal complex components in C. elegans. *Journal of Cell Biology* **219**, e201910043 (2020).
3. Y. Kim, N. Kostow, Abby F. Dernburg, The Chromosome Axis Mediates Feedback Control of CHK-2 to Ensure Crossover Formation in C. elegans. *Developmental Cell* **35**, 247-261 (2015).
4. N. A. Kurhanewicz, D. Dinwiddie, Z. D. Bush, D. E. Libuda, Elevated temperatures cause transposon-associated DNA damage in C. elegans spermatocytes. *Current Biology* **30**, 5007-5017. e5004 (2020).
5. S. Rosu *et al.*, The C. elegans DSB-2 Protein Reveals a Regulatory Network that Controls Competence for Meiotic DSB Formation and Promotes Crossover Assurance. *PLoS genetics* **9**, e1003674 (2013).
6. J. N. Brandt, K. A. Hussey, Y. Kim, Spatial and temporal control of targeting Polo-like kinase during meiotic prophase. *Journal of Cell Biology* **219** (2020).
7. M. P. Colaiacovo *et al.*, Synaptonemal Complex Assembly in C. elegans Is Dispensable for Loading Strand-Exchange Proteins but Critical for Proper Completion of Recombination. *Developmental Cell* **5**, 463-474 (2003).
8. N. Bhalla, D. J. Wynne, V. Jantsch, A. F. Dernburg, ZHP-3 acts at crossovers to couple meiotic recombination with synaptonemal complex disassembly and bivalent formation in C. elegans. *PLoS genetics* **4**, e1000235 (2008).
9. C. M. Phillips *et al.*, HIM-8 Binds to the X Chromosome Pairing Center and Mediates Chromosome-Specific Meiotic Synapsis. *Cell* **123**, 1051-1063 (2005).
10. A. Woglar, A. M. Villeneuve, Dynamic Architecture of DNA Repair Complexes and the Synaptonemal Complex at Sites of Meiotic Recombination. *Cell* **173**, 1678-1691 (2018).
11. T. Nolan, R. E. Hands, S. A. Bustin, Quantification of mRNA using real-time RT-PCR. *Nature Protocols* **1**, 1559-1582 (2006).
